# Development and validation of a dynamic survival prediction model for patients with acute-on-chronic liver failure

**DOI:** 10.1016/j.jhepr.2021.100369

**Published:** 2021-09-29

**Authors:** Ben F.J. Goudsmit, Andries E. Braat, Maarten E. Tushuizen, Minneke J. Coenraad, Serge Vogelaar, Ian P.J. Alwayn, Bart van Hoek, Hein Putter

**Affiliations:** 1 Department of Gastroenterology and Hepatology, Leiden University Medical Center, The Netherlands; 2 Eurotransplant International Foundation, Leiden, The Netherlands; 3 Department of Surgery, Leiden University Medical Center, The Netherlands; 4 Transplant Center, Leiden University Medical Center, The Netherlands; 5 Department of Biomedical Data Sciences, Leiden University Medical Center, The Netherlands

**Keywords:** acute-on-chronic liver failure, liver transplantation, survival prediction, ACLF, acute-on-chronic liver failure, ACLF-JM, acute-on-chronic liver failure joint model, CLIF-C, Chronic Liver Failure-Consortium, INR, international normalized Ratio for the prothrombin time, LT, liver transplantation, MELD-Na, model for end-stage liver disease-sodium, UNOS, United Network for Organ Sharing

## Abstract

**Background & Aims:**

Acute-on-chronic liver failure (ACLF) is usually associated with a precipitating event and results in the failure of other organ systems and high short-term mortality. Current prediction models fail to adequately estimate prognosis and need for liver transplantation (LT) in ACLF. This study develops and validates a dynamic prediction model for patients with ACLF that uses both longitudinal and survival data.

**Methods:**

Adult patients on the UNOS waitlist for LT between 11.01.2016-31.12.2019 were included. Repeated model for end-stage liver disease-sodium (MELD-Na) measurements were jointly modelled with Cox survival analysis to develop the ACLF joint model (ACLF-JM). Model validation was carried out using separate testing data with area under curve (AUC) and prediction errors. An online ACLF-JM tool was created for clinical application.

**Results:**

In total, 30,533 patients were included. ACLF grade 1 to 3 was present in 16.4%, 10.4% and 6.2% of patients, respectively. The ACLF-JM predicted survival significantly (*p* <0.001) better than the MELD-Na score, both at baseline and during follow-up. For 28- and 90-day predictions, ACLF-JM AUCs ranged between 0.840-0.871 and 0.833-875, respectively. Compared to MELD-Na, AUCs and prediction errors were improved by 23.1%-62.0% and 5%-37.6% respectively. Also, the ACLF-JM could have prioritized patients with relatively low MELD-Na scores but with a 4-fold higher rate of waiting list mortality.

**Conclusions:**

The ACLF-JM dynamically predicts outcome based on current and past disease severity. Prediction performance is excellent over time, even in patients with ACLF-3. Therefore, the ACLF-JM could be used as a clinical tool in the evaluation of prognosis and treatment in patients with ACLF.

**Lay summary:**

Acute-on-chronic liver failure (ACLF) progresses rapidly and often leads to death. Liver transplantation is used as a treatment and the sickest patients are treated first. In this study, we develop a model that predicts survival in ACLF and we show that the newly developed model performs better than the currently used model for ranking patients on the liver transplant waiting list.

## Highlights

• ACLF is a dynamic disease that can rapidly change over time, which greatly influences patient survival without LT.
• Currently, the MELD-Na score is used to prioritize patients for LT, but MELD-Na underestimates ACLF disease severity.
• The ACLF joint model (ACLF-JM) was developed to dynamically predict survival.
• The ACLF-JM significantly outperformed the MELD-Na score for the prediction of mortality on the LT waiting list.
• The ACLF-JM can be used online to predict survival in individual patients.

## Graphical abstract

**Figure:**
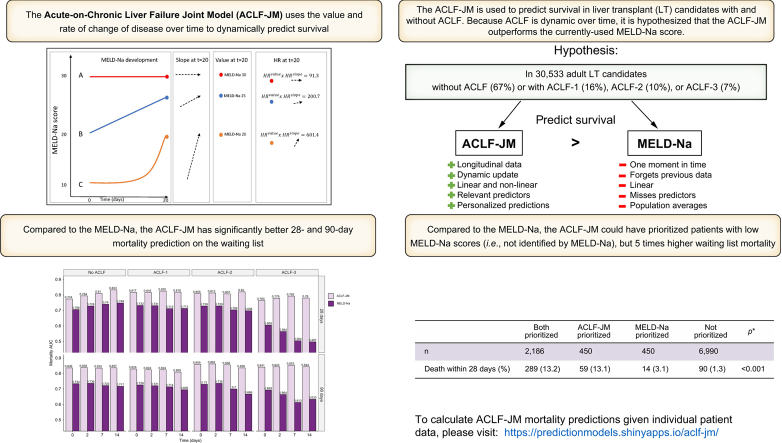

## Introduction

Liver transplantation (LT) is a lifesaving treatment for patients with acute-on-chronic-liver failure (ACLF). ACLF is characterized by an acute deterioration of liver function in patients with chronic liver disease, often started by a precipitating event. ACLF results in the failure of one or more organs and is associated with high short-term mortality.[1], [2], [3] The current model that prioritizes patients for LT, the model for end-stage liver disease-sodium (MELD-Na) score,^4^^,^^5^ underestimates disease severity in ACLF.^6^^,^^7^ This is because MELD-Na does not consider temporal development of single or multiorgan failure(s) (involving the 6 major organs/systems – *i.e.* liver, kidney, brain, coagulation, circulation, and respiration). This underestimation of predicted waitlist mortality results in lower access to transplantation for patients with ACLF.^7^ Sundaram *et al.* showed that ACLF death and waiting list removal rate were highest in ACLF-3 patients with MELD-Na <25.^8^ Given that 20.9% of UNOS LT candidates between 2005-2016 had a form of ACLF,^8^ the overall impact of unequal transplantation access might be substantial.

The MELD-Na score uses one moment in time, *i.e.* the most recent measurement, to predict outcome.^4^^,^^5^ It therefore ignores previous data that could be valuable for survival estimation. However, ACLF is a dynamic disease with a clinical course that can change within days, resulting in very different outcomes.^9^^,^^10^ Thus, there is a need for prediction models that estimate ACLF survival based on disease development over time.^7^ The Chronic Liver Failure-Consortium organ failure (CLIF-C OF) and CLIF-C ACLF scores were developed for this purpose and showed better performance than the MELD-Na score.^3^^,^^6^ However, they also assessed only a single moment in time. A joint model (JM) is a novel prediction model that simultaneously uses longitudinal and survival data.^11^ It approximates changing disease severity over time and uses this for survival prediction.^12^ JMs have shown superior predictive performance over Cox models.[12], [13], [14] However, they have not been applied to ACLF.

We hypothesized that using disease development over time to dynamically predict prognosis could improve survival prediction in patients with ACLF. Much like a clinician, we aimed to use disease severity and its rate of change to predict outcome. We believe this is warranted in ACLF, because of the dynamic nature of the disease and the current underestimation of mortality by MELD-Na.^7^^,^^9^^,^^10^ Therefore, we constructed and validated a multivariate prediction model for survival prediction in patients with ACLF: the ACLF-JM. We investigated the performance of ACLF-JM for 28- and 90-day survival prediction in the United Network for Organ Sharing (UNOS) registry and compared its performance to the MELD-Na score. We also investigated whether the ACLF-JM could identify patients in whom MELD-Na underestimates mortality. For easy clinical application, an online ACLF-JM tool was developed for dynamic survival prediction in patients with ACLF.

## Materials and methods

The TRIPOD statement was used for the development and validation of this multivariate prediction model.^15^

### Study population

Data on LT candidates was requested from the UNOS. We included adult (≥18 years) patients listed for a first LT between January 11, 2016 (after MELD-Na implementation) and December 31, 2019. We excluded candidates with acute liver failure and hepatocellular carcinoma at baseline. Data were used from first active listing until the earliest of patient death, transplantation, removal or censor at December 31, 2019. Death was defined both as death while listed and removal for being too sick to transplant.^8^ If patients received exception points or a status 1 (*i.e.* high urgency status) after first listing, they were censored from that date. MELD-Na data was missing in 0.05%, therefore complete-case analysis was done. Missing values for the predictors life support dependency (variable CAN_LIFE_SUPPORT, 0.00009% missing) and spontaneous bacterial peritonitis (CAN_BACTERIA_PERIT, 0.005% missing) were set to ‘no’.

### Identification of ACLF

Baseline ACLF was defined according to the to the European Foundation for the Study of Chronic Liver Failure (EF Clif) criteria.^3^ Specifically, liver failure was defined as serum bilirubin ≥12 mg/dl, kidney failure as serum creatinine ≥2.0 mg/dl or renal replacement therapy, cerebral failure as presence of hepatic encephalopathy grade 3-4, coagulation failure as international normalized ratio (INR) ≥2.5. Like other authors that used UNOS data, we used mechanical ventilation as a surrogate for respiratory failure, since data on PaO_2_/FiO_2_ were not available. Also, life support dependency was used to designate circulatory failure.^6^^,^^8^^,^^10^^,^^16^

### Development of the ACLF-JM

**Fig. 1:**
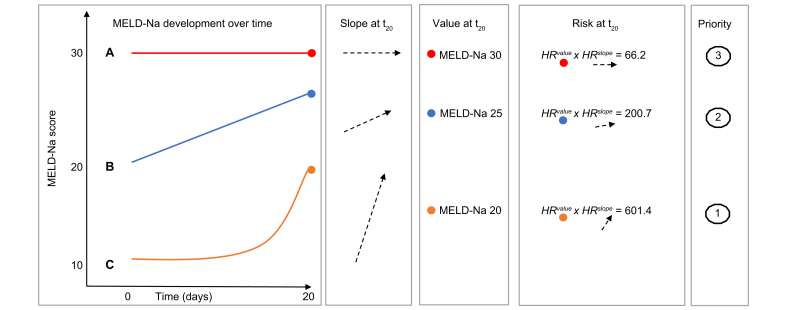
Joint model use of value and slope (rate of change). For 3 hypothetical patients A, B and C, the 20-day MELD-Na development is shown. After 20 days, patient A has a MELD-Na score of 30 and is thus prioritized by the current allocation system. However, the ACLF-JM uses both the estimated value (measured MELD-Na score) and slope (rate of change) at time=20 for survival prediction. Calculation of the HRs shows that the ACLF-JM gives patient C the greatest risk of death, because of the fast increase in MELD-Na scores (positive slope). See supplement 4 for the precise explanation and calculation. ACLF, acute-on-chronic liver failure; ACLF-JM, acute-on-chronic liver failure joint model; HR, hazard ratio; MELD-Na, model for end-stage liver disease-sodium.

Data were randomly split into a training (67% of the patients) and a testing (33%) set, for model development and validation, respectively. The ACLF-JM consists of 2 parts: a longitudinal (mixed-effect) and survival (Cox proportional hazards) model. Mixed-effect models were used because they estimate disease development over time as a continuous trajectory and can model both linear (chronic, stable disease) and non-linear (fast deterioration in ACLF) developments. See Fig. S4 for an illustration. Thus, repeated measurements of MELD-Na scores were modelled with mixed-effects. Additional predictors were used to correct the longitudinal data. To start, 50 candidate variables were assessed (Table S2). We excluded some variables *a priori*, because they referred to pediatric recipients, exclusion criteria, or donor characteristics. Variable relation to mortality was studied in univariate analysis and then variables were backwards selected for multivariate Cox analysis. The final variables included in the model contributed most significantly besides those used for ACLF scoring through EF Clif criteria (serum bilirubin, creatinine, renal replacement therapy, encephalopathy grade, INR, mechanical ventilation, and life support dependency). Thus, we additionally corrected for candidate age (years), sex (male/female), life support dependency (yes/no), presence of bacterial peritonitis (yes/no), presence of cirrhosis (alcohol-induced, hepatitis-C virus, non-alcoholic steatohepatitis [NASH] or other cirrhosis) (yes/no) and CLIF-C OF score (No ACLF or ACLF grade 1 to 3) (Table S1). Next, a Cox proportional hazards model was constructed for waiting list mortality, using the same predictors as the mixed-effect model. Then, the ACLF-JM was constructed by joint-modelling the longitudinal (mixed-effect) and survival (Cox) model.^17^ A key feature is that the ACLF-JM uses both the estimated MELD-Na value and the rate of change in MELD-Na (the slope of the decrease/increase) over time for survival prediction. For clarity, these concepts of value and slope are illustrated in Fig. 1.

### Validation of the ACLF-JM

Next, the prediction performance of the ACLF-JM was compared to the MELD-Na at various points in time in the separate testing data. Specifically, predictions were assessed at baseline and after a follow-up of 48 hours, 7 days and 14 days (similar to the validation study of the CLIF-C OF).^6^ Outcomes were 28-day and 90-day survival. For both the ACLF-JM and MELD-Na Cox model, the area under the receiver-operating characteristic curve (AUC) and prediction errors were calculated and compared (see supplement 3 for detailed information). These measures and their 95% CIs and *p* values were calculated using the R package JM and bootstrapping.^17^

### ACLF-JM impact on the transplantation waiting list

Next, we assessed the possible effect of using the ACLF-JM instead of MELD-Na to estimate mortality and subsequently prioritize patients for LT. This was of interest, because patients with ACLF are likely underserved in the current LT allocation.^7^ To assess possible differences in MELD-Na and ACLF-JM waitlist prioritization of patients, we followed patients from baseline until day 28.^6^ Within this period, each time a liver graft was offered, patients were ranked twice from most to least ill based on their estimated survival without transplant. One ranking was made with the ACLF-JM predictions and one based on MELD-Na. Thus, for each model, patients were ranked 2,636 times, *i.e.* the total number of available liver grafts within the first 28 days. After a liver graft offer, the transplanted patient was removed from the waiting list. We assumed that the highest ranked patients were transplanted, which is not necessarily true, and thus that the number of available transplants in the first 28 days represented the threshold of receiving transplantation. We then assessed which patients were prioritized according to what model. After 28 days and 2,636 rankings, patients were stratified into 4 groups: those who are prioritized and possibly transplanted within 28 days according to both scores, those who are prioritized by either the ACLF-JM or MELD-Na score (but not by both) and those who are not prioritized by either. We also assessed the characteristics of the differently prioritized patients, to see why they were prioritized differently.

### Clinical application of the ACLF-JM

Lastly, an online version of the ACLF-JM was created (https://predictionmodels.shinyapps.io/aclf-jm/), which allows clinicians to assess ACLF-JM survival predictions for their individual patient(s). Plots can be created from these dynamic predictions, to show the updating survival estimate for every new available measurement during follow-up. For an instruction manual, see supplement 1 and 2. All statistical analyses were performed using R v4.0.0 (R Foundation for Statistical Computing, Vienna, Austria).

## Results

### Study population

In total, we included 30,533 patients with 249,030 measurements. Table 1 shows the baseline characteristics of the study population. ACLF at baseline was seen in 33.3% of the patients; 15.9% had ACLF grade 1, 10.3% had grade 2 and 7.1% had grade 3. In these patients, liver (47.2%) and kidney (63.6%) failure were the most common. With increasing ACLF grade, median [IQR] age decreased, ranging from 59 [52-64] (no ACLF) to 53 [43-60] years (ACLF-3). Most patients were male (no ACLF: 65.0%, ACLF: 60%) and had alcohol-related liver disease (no ACLF 25.8%, ACLF 40%). For ACLF grades 0 to 3, median [IQR] MELD-Na scores at listing were 15 [10-22], 27 [23-31], 33 [29-37] and 37 [31-42]. Average time on the waiting list was 150 days for patients without ACLF, 89 for ACLF grade 1, 24 for grade 2 and 10 days for grade 3. Cumulative incidence plots showed significantly higher death and transplantation rates in patients with ACLF (Fig. S1). At the end of follow-up, 10.9% of the patients without ACLF died. For patients with ACLF grade 1 to 3, death rates were 16.7%, 14.3% and 22.4%, respectively.

**Table 1:** **Baseline characteristics**.

Baseline characteristics of UNOS liver transplantation candidates between 2016 to 2019 (n = 30,533)							
	No ACLF	ACLF (any grade)	*p* value†	ACLF-1	ACLF-2	ACLF-3	*p* value‡
Number of patients (%)	20,384 (66.7)	10,149 (33.3)		4,843 (15.9)	3,147 (10.3)	2,159 (7.1)	
Age (median [IQR])	59 [52, 64]	55 [47, 62]	<0.001	58 [50, 64]	53 [44, 60]	53 [43, 60]	<0.001
Male gender	13,240 (65)	6094 (60)	<0.001	2,905 (60.0)	1,919 (61.0)	1,270 (58.8)	<0.001
BMI (median [IQR])	28 [25, 33]	29 [25, 33]	<0.001	28 [24, 33]	29 [25, 34]	30 [26, 35]	<0.001
Days waiting (median [IQR])	58 [14, 193]	12 [4, 40]	<0.001	27 [9, 93]	8 [4, 20]	5 [3, 10]	<0.001
Status after waiting			<0.001				<0.001
Censored (December 31, 2019)	986 (4.8)	185 (1.8)		129 (2.7)	43 (1.4)	13 (0.6)	
Deceased	2,229 (10.9)	1,745 (17.2)		810 (16.7)	451 (14.3)	484 (22.4)	
Transplanted	8,681 (42.6)	7,247 (71.4)		3,187 (65.8)	2,472 (78.6)	1,588 (73.6)	
Removed	8,488 (41.6)	972 (9.6)		717 (14.8)	181 (5.8)	74 (3.4)	
Grouped cause of disease (%)			<0.001				<0.001
Cirrhosis HCV∗	3,084 (15.1)	917 (9.0)		556 (11.5)	205 (6.5)	156 (7.2)	
NASH∗	4,359 (21.4)	1,969 (19.4)		1,184 (24.4)	500 (15.9)	285 (13.2)	
Cirrhosis alcohol-induced∗	5,252 (25.8)	4,057 (40.0)		1,680 (34.7)	1,431 (45.5)	946 (43.8)	
Cirrhosis other∗	2,976 (14.6)	1,778 (17.5)		682 (14.1)	616 (19.6)	480 (22.2)	
Cholestatic disease	1,810 (8.9)	612 (6.0)		343 (7.1)	182 (5.8)	87 (4.0)	
Metabolic disease	408 (2.0)	245 (2.4)		112 (2.3)	81 (2.6)	52 (2.4)	
Malignant/benign tumor	2,119 (10.4)	266 (2.6)		194 (4.0)	42 (1.3)	30 (1.4)	
Other	376 (1.8)	305 (3.0)		92 (1.9)	90 (2.9)	123 (5.7)	
MELD-Na score (median [IQR])	15 [10, 20]	30 [25, 35]	<0.001	27 [22, 31]	33 [29, 37]	37 [31, 42]	<0.001
Bacterial peritonitis (%)	1,560 (7.7)	1,533 (15.1)	<0.001	643 (13.3)	508 (16.1)	329 (17.4)	<0.001
Failure organ/system (%)							
Liver	540 (2.6)	4,789 (47.2)	<0.001	1,018 (21.0)	2,007 (63.8)	1,764 (81.7)	<0.001
Kidney	0 (0.0)	6,457 (63.6)	<0.001	2,958 (61.1)	1,717 (54.6)	1,782 (82.5)	<0.001
Coagulation	254 (1.2)	3,699 (36.4)	<0.001	667 (13.8)	1,613 (51.3)	1,419 (65.7)	<0.001
Cerebral	806 (4.0)	2,095 (20.6)	<0.001	164 (3.4)	697 (22.1)	1,234 (57.2)	<0.001
Circulatory	22 (0.1)	1,193 (11.8)	<0.001	36 (0.7)	221 (7.0)	936 (43.4)	<0.001
Respiratory	0 (0.0)	662 (6.5)	<0.001	0 (0.0)	39 (1.2)	623 (28.9)	<0.001

*p* values were derived with non-parametric and chi-square tests.

NASH, non-alcoholic steatohepatitis, MELD-Na, model for end-stage liver disease-sodium

∗ These patients received cirrhosis = 1 in the JM

† Comparison between patients with ACLF and without ACLF (chi-square and ANOVA tests).

‡ Comparison between ACLF grades (chi-square and ANOVA tests).

### Model properties

The ACLF-JM is summarized by the equation: $Hazard\;Ratio\;death_{t}=1.15^{MELDNa_{value\;t}}*1.02^{MELDNa_{slope\;t}}*1.38^{age}*0.75^{female\;gender}*0.95^{cirrhosis}\;*\left(if:1.06^{ACLF1}\right)\;*\left(if:1.98^{ACLF2}\right)\;*\;\left(if:5.90^{ACLF3}\right)\;*1.18^{SBP}*1.35^{life\_support}.$ The ACLF-JM estimates the MELD-Na value and slope at a given timepoint and calculates the hazard ratio of death. For each MELD-Na point increase, the risk of death at 1 year increases by 15% (95% CI 14-16). For every 1-point increase in slope, *i.e.* acceleration of disease increase, the mortality risk increases by 2% (95% CI 1-2). Of course, in clinical practice, disease severity often changes more rapidly, especially for patients with ACLF. A more intuitive illustration of the effect of MELD-Na value and slope is provided in Fig. 1, where 3 hypothetical cases are shown. The example calculation (details in supplement 4) shows that considering the rate of change (slope) in disease severity adds important information. Considering both MELD-Na value and slope would give priority to patient C (MELD-Na score 20, accelerating disease severity), whereas using the current MELD-Na-based allocation would prioritize patient A (MELD-Na 30, stable disease).

### Model validation

**Fig. 2:**
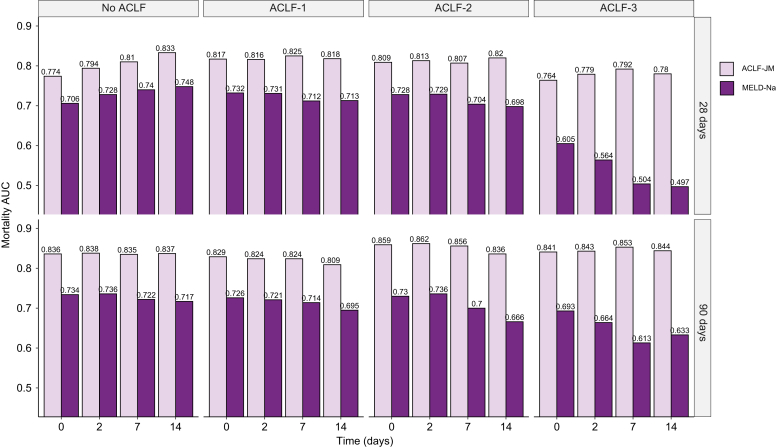
AUCs of the ACLF-JM and MELD-Na, stratified per ACLF grade. The AUCs for 28- and 90-day mortality prediction of the ACLF-JM and the MELD-Na, stratified for ACLF severity. ACLF, acute-on-chronic liver failure; ACLF-JM, acute-on-chronic liver failure joint model; AUC, area under receiver-operating characteristic curve MELD-Na, model for end-stage liver disease-sodium.

The ACLF-JM prediction performance was validated in separate testing data. Table 2 shows the 28- and 90-day prediction performance of the ACLF-JM and MELD-Na, stratified for patients with and without ACLF, at baseline and during follow-up. For all time points and studied outcomes, the JM performance was significantly better than MELD-Na. At baseline in patients with ACLF, the ACLF-JM AUC was 0.875 (95% CI 0.840-0.909) and MELD-Na AUC was 0.780 (95% CI 0.737-0.823). During follow-up, AUCs of both models declined to 0.833 (0.799-0.868) and 0.719 (0.677-0.761) respectively, which is still excellent for the ACLF-JM and respectable for the MELD-Na (also see Fig. S2A and S3). Fig. 2 shows that with increasing ACLF grade, JM performance remains significantly better than the declining MELD-Na (also see Table S3 and Fig. S3). The performance of the ACLF-JM was particularly good for 90-day mortality prediction in patients with ACLF grade 3, with AUCs ranging from 0.841 to 0.853, contrasting with the MELD-Na AUCs of between 0.613 and 0.693. AUCs for MELD-Na were (almost) equal when predicting 28-day mortality in patients with ACLF-3, ranging from 0.497 to 0.605. Importantly, the ACLF-JM also better estimated risks, *i.e.* is better calibrated, than the MELD-Na (Fig. S2B). With increasing ACLF grade, prediction errors were improved up to 37.6% (Fig. S3B). An accurate model is important for clinical decision making, because decisions are often based on risks.^18^

**Table 2:** **AUCs of the ACLF-JM and MELD-Na**.

Mortality prediction AUC of the ACLF-JM *vs*. the MELD-Na in patients with and without ACLF, at baseline and during follow-up								
	**ACLF**				**No ACLF**			

**28-day mortality**	**ACLF-JM**	**95% CI**	**MELD-Na**	**95% CI**	**ACLF-JM**	**95% CI**	**MELD-Na**	**95% CI**

Baseline	0.871	0.844-0.898	0.788	0.754-0.822	0.774	0.717-0.831	0.706	0.643-0.769
48 hours	0.871	0.844-0.898	0.786	0.752-0.820	0.794	0.741-0.847	0.728	0.668-0.788
7 days	0.862	0.833-0.890	0.753	0.716-0.789	0.810	0.761-0.859	0.740	0.684-0.796
14 days	0.840	0.803-0.878	0.731	0.685-0.777	0.833	0.788-0.879	0.748	0.694-0.802

	**ACLF**				**No ACLF**			

**90-day mortality**	**ACLF-JM**	**95% CI**	**MELD-Na**	**95% CI**	**ACLF-JM**	**95% CI**	**MELD-Na**	**95% CI**

Baseline	0.875	0.840-0.909	0.780	0.737-0.823	0.836	0.807-0.865	0.734	0.700-0.768
48 hours	0.870	0.837-0.903	0.777	0.735-0.818	0.838	0.810-0.867	0.736	0.703-0.770
7 days	0.861	0.832-0.891	0.755	0.717-0.792	0.835	0.806-0.864	0.722	0.687-0.757
14 days	0.833	0.799-0.868	0.719	0.677-0.761	0.837	0.809-0.865	0.717	0.682-0.752

All AUCs differed significantly (*p* <0.001)

ACLF, acute-on-chronic liver failure; ACLF-JM, acute-on-chronic liver failure joint model; AUC, area under receiver-operating characteristic curve; MELD-Na, model for end-stage liver disease-sodium.

### ACLF-JM impact on the transplantation waiting list

**Fig. 3:**
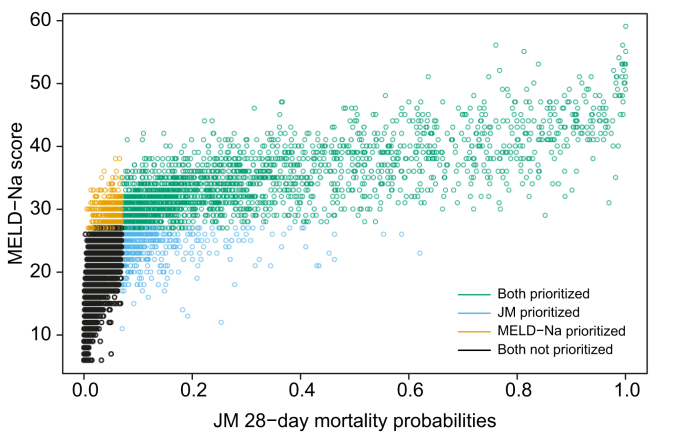
Correlation plot of the ACLF-JM and MELD-Na at 28 days. The correlation plot of MELD-Na score and ACLF-JM survival predictions. Patients are stratified in 4 groups: orange and blue patients would have been prioritized differently under either the ACLF-JM or MELD-Na. Blue patients had a 4x higher 28-day waiting list mortality than orange patients. ACLF, acute-on-chronic liver failure; ACLF-JM, acute-on-chronic liver failure joint model; MELD-Na, model for end-stage liver disease-sodium.

To study the difference in survival prediction and subsequent allocation priority between the ACLF-JM and the MELD-Na, patients were followed-up for the first 28 days. In total, 2,636 transplants were performed within this period. Fig. 3 shows the correlation plot between MELD-Na scores and ACLF-JM mortality estimates after 28 days of waiting list follow-up. For 2,186 patients (in green), transplantation priority was given according to both the ACLF-JM and MELD-Na, as estimated mortality without LT was highest. More interestingly, 450 patients (in blue) could possibly have been prioritized by the ACLF-JM, but not by MELD-Na. Importantly, although these patients had lower median MELD-Na scores, they also had 4-fold higher 28-day mortality rates, *i.e.* 13.1% *vs.* 3.1% (Table 3). Compared to the 450 MELD-Na-prioritized patients (orange), ACLF-JM-prioritized patients were older, more often female, had lower ACLF-1 rates, more NASH, less alcohol-induced liver disease and were more often dependent on life support. After 28 days, 190 patients were delisted due to increased disease severity. In these patients, the survival prediction AUCs (95% CI) for the ACLF-JM and MELD-Na score were 88.0 (85.1-90.9) and 82.5 (79.0-85.9), respectively (Fig. S6).

**Table 3:** **Characteristics of prioritized patients according to ACLF-JM and MELD-Na**.

Characteristics of patients prioritized differently for liver transplantation within 28 days					
	Both prioritized	ACLF-JM prioritized	MELD-Na prioritized	Not prioritized	*p* value∗
n	2,186	450	450	6,990	
Age (median [IQR])	56.0 [47.0, 62.0]	62.0 [55.0, 67.0]	50.0 [42.0, 56.8]	59.0 [52.0, 64.0]	<0.001
Male sex (%)	1,336 (61.1)	175 (38.9)	326 (72.4)	4,552 (65.1)	<0.001
Death within 28 days (%)	289 (13.2)	59 (13.1)	14 (3.1)	90 (1.3)	<0.001
ACLF (%)					<0.001
No ACLF	172 (7.9)	191 (42.4)	162 (36.0)	6,155 (88.1)	
ACLF-1	585 (26.8)	95 (21.1)	248 (55.1)	720 (10.3)	
ACLF-2	792 (36.2)	91 (20.2)	39 (8.7)	105 (1.5)	
ACLF-3	637 (29.1)	73 (16.2)	1 (0.2)	10 (0.1)	
Disease					<0.001
Cirrhosis HCV	165 (7.5)	31 (6.9)	39 (8.7)	1,099 (15.7)	
NASH	392 (17.9)	147 (32.7)	61 (13.6)	1,479 (21.2)	
Cirrhosis alcohol-induced	964 (44.1)	130 (28.9)	235 (52.2)	1,768 (25.3)	
Cirrhosis other	416 (19.0)	68 (15.1)	55 (12.2)	988 (14.1)	
Cholestatic disease	104 (4.8)	41 (9.1)	36 (8.0)	638 (9.1)	
Metabolic disease	56 (2.6)	5 (1.1)	13 (2.9)	135 (1.9)	
Malignant/benign tumor	39 (1.8)	11 (2.4)	7 (1.6)	734 (10.5)	
Other	50 (2.3)	17 (3.8)	4 (0.9)	149 (2.1)	
MELD (median [IQR])	34.0 [29.0, 39.0]	24.0 [21.0, 28.0]	26.0 [23.0, 29.0]	15.0 [11.0, 19.0]	<0.001
MELD-Na (median [IQR])	33.0 [30.0, 38.0]	25.0 [23.0, 26.0]	28.0 [27.0, 30.0]	15.0 [10.0, 20.0]	<0.001
Life support dependent	291 (13.3)	84 (18.7)	3 (0.7)	50 (0.7)	<0.001

Clarification: JM-prioritized patients are not prioritized by MELD-Na, and vice versa.

ACLF, acute-on-chronic liver failure; ACLF-JM, acute-on-chronic liver failure joint model; MELD(-Na), model for end-stage liver disease(-sodium); NASH, non-alcoholic steatohepatitis.

∗ Difference tested between ACLF-JM-prioritized and MELD-Na-prioritized patients (chi-square and ANOVA tests).

### Clinical application of the ACLF-JM

After constructing and validating the ACLF-JM in this large cohort, an online application was developed, which allows clinicians to easily calculate individual patient survival probabilities based on the ACLF-JM. Available at: https://predictionmodels.shinyapps.io/aclf-jm/. Excel files with repeated MELD-Na measurements can be uploaded into this tool, to generate dynamic survival predictions during follow-up. The ACLF-JM simulates individual patient data to calculate personalized predictions. See supplement 1 for precise instructions for the data upload and supplement 2 for a step-by-step manual.

## Discussion

In this study, we developed and validated the ACLF-JM prediction model, to estimate survival of patients with ACLF. We report several important findings. First, both current and past disease severity and its rate of change are strongly associated with survival in ACLF. Second, by using these data, the ACLF-JM gives excellent prediction performance, even in ACLF-3, and significantly outperforms MELD-Na. Third, the ACLF-JM could have prioritized patients with low median MELD-Na scores, *i.e*., not identified by MELD-Na, but with 4-fold higher mortality rates than MELD-Na-prioritized patients. Fourth, the ACLF-JM can be clinically applied online to estimate and visualize patient-specific survival, which can be updated with every new measurement.

ACLF disease severity is dynamic and can change rapidly. During the first week, disease severity changes for most patients, resulting in different survival outcomes.^9^^,^^10^ The current liver allocation system does not consider change, as it uses only the most recent measurement for survival prediction and ignores previous data. Moreover, survival is estimated based on the MELD-Na score, which ignores relevant factors for ACLF and therefore underestimates mortality.^7^^,^^8^ Hernaez *et al.* showed that mortality was higher than expected in low MELD-Na score patients. They also showed that, despite their high(er) ACLF grade, these low MELD-Na patients were often not considered for LT.^7^ Interestingly, Hernaez *et al.* stated that "Future research should also focus on developing and validating prognostic scores that incorporate dynamic changes in patients clinical course", *i.e.* the goal of this study. Sundaram *et al.* showed that ACLF death and removal rate did not correlate well with the MELD-Na score, as mortality rates were highest in ACLF-3 patients with MELD-Na <25.^8^ In this study, ACLF was present in 33.3% (Table 1) of the patients. As a result, the MELD-Na underestimation of ACLF disease severity could be substantial, which possibly leads to unequal treatment access and surplus mortality.^7^ Therefore, the ACLF-JM was developed to predict ACLF patient survival based on disease development over time. The model provides several important improvements over the MELD-Na score (Table S4).^19^ Most importantly, predictions are based on all available previous data and update for every new measurement.^20^ Predictions should update based on accumulating evidence, because ACLF is a dynamic disease. The ACLF-JM can handle varying measurements per patient and varying time between measurements, which is likely for waiting list data over time. At minimum, 1 measurement is required per patient to give a survival prediction. With more available measurements over time, increasingly accurate estimates can be made. The ACLF-JM also considers both the value of disease severity and the rate at which disease severity is changing (Fig. 1). It uses more nuanced aspects of ACLF disease development to predict survival. Thus, like a clinician, past and current disease developments are used to estimate patient prognosis. Updating prognosis is important in ACLF, as disease can increase fast and non-linearly (*e.g.* exponentially).^1^^,^^3^ ACLF-JM survival predictions could therefore be used to aid clinical decision making for patients with ACLF on the waiting list for LT, as current models result in unequal transplantation access and post-LT survival rates.^8^^,^^10^^,^^16^ Furthermore, In this cohort, we showed that ACLF-JM prioritization identified patients with low MELD-Na scores, but high mortality (Table 3). Mortality is underestimated in these patients and subsequently they receive a lower priority for LT. Since patients with ACLF benefit from fast LT,^16^ use of the ACLF-JM for the evaluation of prognosis could perhaps help to resolve the underestimation of waiting list mortality in patients with ACLF.^7^

The ACLF-JM showed excellent performance for the prediction of short-term survival at baseline and with increasing follow-up. Increasing ACLF grade did not lead to a decrease in predictive accuracy. This is important, because risk of death and need for LT should be reliably estimated in the sickest patients. Our data showed that both the ACLF-JM and MELD-Na AUCs declined with increasing follow-up. This is likely due to population changes, *i.e.* the sickest patients die or are transplanted first and less patients remain with increasing follow-up.^21^ Also, with increasing disease severity, generally a shorter follow-up period is available. The ACLF-JM approximation of disease does not depend on the number of measurements per patient, because it estimates disease over time as a continuous trajectory (Fig. S4). This is important, because frequency of measurement confounded previous (Cox-based) survival predictions for patients in need of LT.^22^ The ACLF-JM performed comparably or sometimes even better than the CLIF-C OF score.^6^ This could possibly indicate that ACLF-JM performance was adequate for clinical application. Because the UNOS registry does not contain data on white blood cell counts, CLIF-C ACLF scoring was not possible in this study. ACLF-JM performance could however be externally validated in the cohorts used to construct the CLIF-C scores.^6^

The differences in waiting list prioritization between the ACLF-JM and MELD-Na were investigated for the first 28 days.^6^ The results of this prioritization naturally depend on the chosen time period and we did not represent the complex reality of liver allocation. However, the goal was to illustrate how the ACLF-JM prioritized differently from the MELD-Na, because of its inherent use of disease development and rate of change over time. After training and ascertaining excellent performance, an online ACLF-JM tool was created for clinical use. Especially in ACLF, both the patient and treating clinician benefit from patient-specific modelling, which shifts the focus of prediction from the population to the individual patient level. Jalan *et al.* already stated that there is a need for models that “update on a daily basis providing additional prognostic information”, and that “currently, no validated evidence-based tools guide the decision-making”.^6^ The ACLF-JM meets these demands and more, with excellent performance leading to personalized prediction, readily available online for any clinician.

A limitation is that longitudinal MELD-Na measurements are not best to model ACLF disease development, as they can underestimate ACLF disease severity.^7^ Ideally, longitudinal CLIF-C ACLF score data would be available in the UNOS registry, but currently missing leucocyte counts prevent CLIF-C ACLF scoring. Further information on lactate levels and bacterial infection would be valuable to register for LT candidates.^23^ The MELD-Na was one of the few consistently available longitudinal measurements, which allowed for analysis on a large scale and comparison to previous studies. The retrospective analysis of large databases also has several disadvantages. Misclassification of disease severity could introduce bias, *e.g.* subjective scoring of ascites and encephalopathy. Also, surrogate markers, suggested by authors of other large UNOS ACLF analyses, were used for ventilatory and circulatory failure.^6^^,^^8^^,^^10^^,^^16^ For example, mechanical ventilation was used as replacement for respiratory failure, it is however very well possible that a patient with respiratory failure did not receive mechanical ventilation, or vice versa. Despite these shortcomings, the ACLF-JM showed excellent performance with increasing disease severity (ACLF grade).

ACLF survival is dynamically predicted by the ACLF-JM prediction model, using both longitudinal and survival data. Updating prognosis on new measurements is important, as ACLF is a dynamic disease. The ACLF-JM prediction performance was excellent in this cohort, even in patients with ACLF-3. The ACLF-JM could therefore be used as a tool for the personalized evaluation of prognosis and clinical decision making in patients with ACLF.

## Financial support

The manuscript was not prepared by or funded in any part by a commercial organization. No financial support or grants were used for the preparation of this manuscript.

## Authors’ contributions

BG, HP, BH, AB contributed to the design of the study. BG contributed to data acquisition. BG and HP contributed to data analysis. All authors were involved in interpretation of the data, drafting, and revising the manuscript. All authors approved the final version of the manuscript for submission.

## Data availability statement

The data are publicly available from OPTN/UNOS, but the authors are not able to share the data due to restrictions in the data use agreement.

## Conflicts of interest

The authors of this manuscript have no conflict of interest to disclose.

Please refer to the accompanying ICMJE disclosure forms for further details.
