# Supplementary material for: Development and validation of a dynamic survival prediction model for patients with acute-on-chronic liver failure
Source: JHEP Rep. 2021 Sep 29;3(6):100369. doi: 10.1016/j.jhepr.2021.100369 (PMC8570961; doi:10.1016/j.jhepr.2021.100369)
Supplement: Multimedia component 1 [file mmc1.pdf]

Development and validation of a dynamic survival prediction model for patients with acute-on-chronic liver failure

Ben F.J. Goudsmit, Andries E. Braat, Maarten E. Tushuizen, Minneke J. Coenraad, Serge Vogelaar, Ian P.J. Alwayn, Bart van Hoek, Hein Putter

Table of contents

Supplement 1.. 2

Supplement 2. 4

Supplement 3. 12

Supplement 4. 17

Supplementary figure legends 18

Fig. S1 20

Fig. S2 21

Fig. S3 23

Fig. S4 25

Fig. S5 26

Fig. S6 27

Table S1 28

Table S2 29

Table S3 31

Table S4 32

Supplement 1: instructions for ACLF-JM upload data preparation.

The online ACLF-JM tool requires the user to upload patient data in an Excel file, .csv format. To make the predictions work, data needs to have the same properties as the example below. Please use the example below as template (i.e. column names and data variables).
Specifically: time_of_measurement and meldna are allowed to vary, with new values in every row. All the other variables should be kept constant, i.e. fill in the first row and then copy the contents until the last row of data. Ideally, these variables should be extracted from the UNOS or SRTR database, or from your own hospital with the help of data managers.

id	time_of_ measurement	meldna	time_of_last_ measurement	death	age	femalesex	cirrhosis_present	aclf_grade	sbp_present	life_support_dependent
1	0	27	51	0	50	0	0	ACLF-1	0	0
1	5	24	51	0	50	0	0	ACLF-1	0	0
1	6	24	51	0	50	0	0	ACLF-1	0	0
1	11	26	51	0	50	0	0	ACLF-1	0	0
1	13	26	51	0	50	0	0	ACLF-1	0	0
1	14	27	51	0	50	0	0	ACLF-1	0	0
1	21	25	51	0	50	0	0	ACLF-1	0	0
1	24	25	51	0	50	0	0	ACLF-1	0	0
1	31	25	51	0	50	0	0	ACLF-1	0	0

Instructions:

- In the ID column: enter a random ID number (e.g. 1).
- In the time_of_measurement column: set the day of measurement, e.g. if you followed a patient from day 0 (start measurements) to day 50 (current date). If your measurements are taken on a specific date, please visit the following link to calculate the date difference in days: https://support.microsoft.com/en-us/office/calculate-the-difference-between-two-dates-8235e7c9-b430-44ca-9425-46100a162f38
- In the meldna column: enter the repeated measured MELD-Na scores of your patient.
- In the time_of_last_measurement column: enter the last time (in days) your patient was measured. Typically, it is the date difference between the current date and start of follow-up, e.g. 51 days.

- In the death column: enter 1 if your patient died at the time_of_last_measurement, if not: enter 0.
- In the age column: enter the age of your patient in years.
- In the femalesex column: enter 1 if your patient is female, if not: enter 0.
- In the aclf_grade column: enter the CLIF-C OF score, which can be calculated at: https://www.efclif.com/scientific-activity/score-calculators/clif-c-aclf The possible levels are: "No ACLF", "ACLF-1","ACLF-2" or "ACLF-3".
- In the sbp_present column: enter 1 if your patient has bacterial peritonitis, if not: enter 0.
- In the life_support_dependent colum: enter 1 if your patient is on life support, if not: enter 0.

Save the file (as .csv).

Then upload in the online application and predict survival for you patient.

Supplement 2: Step-by-step instruction manual

Upon opening the link, the following interface will show. Please press the “Browse” button to upload patient data (.csv file).

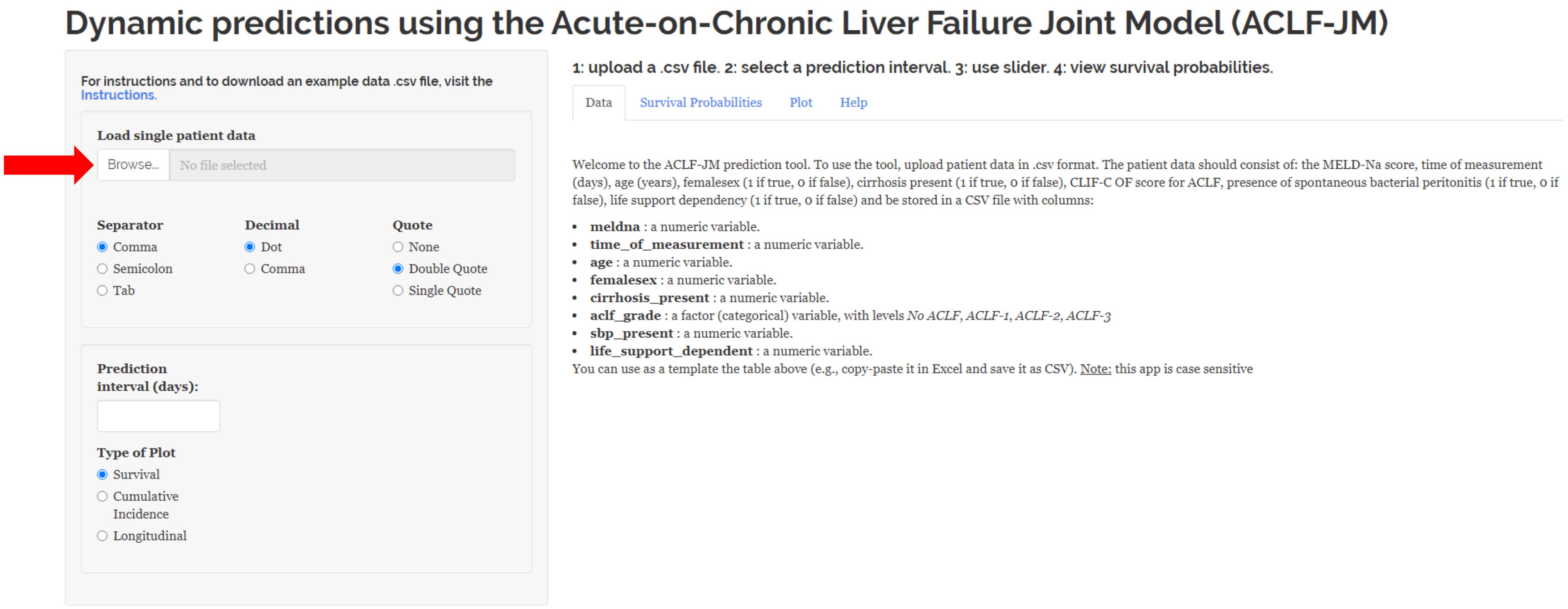

Open the local .csv file from your computer. Below, the supplied example data file is loaded.

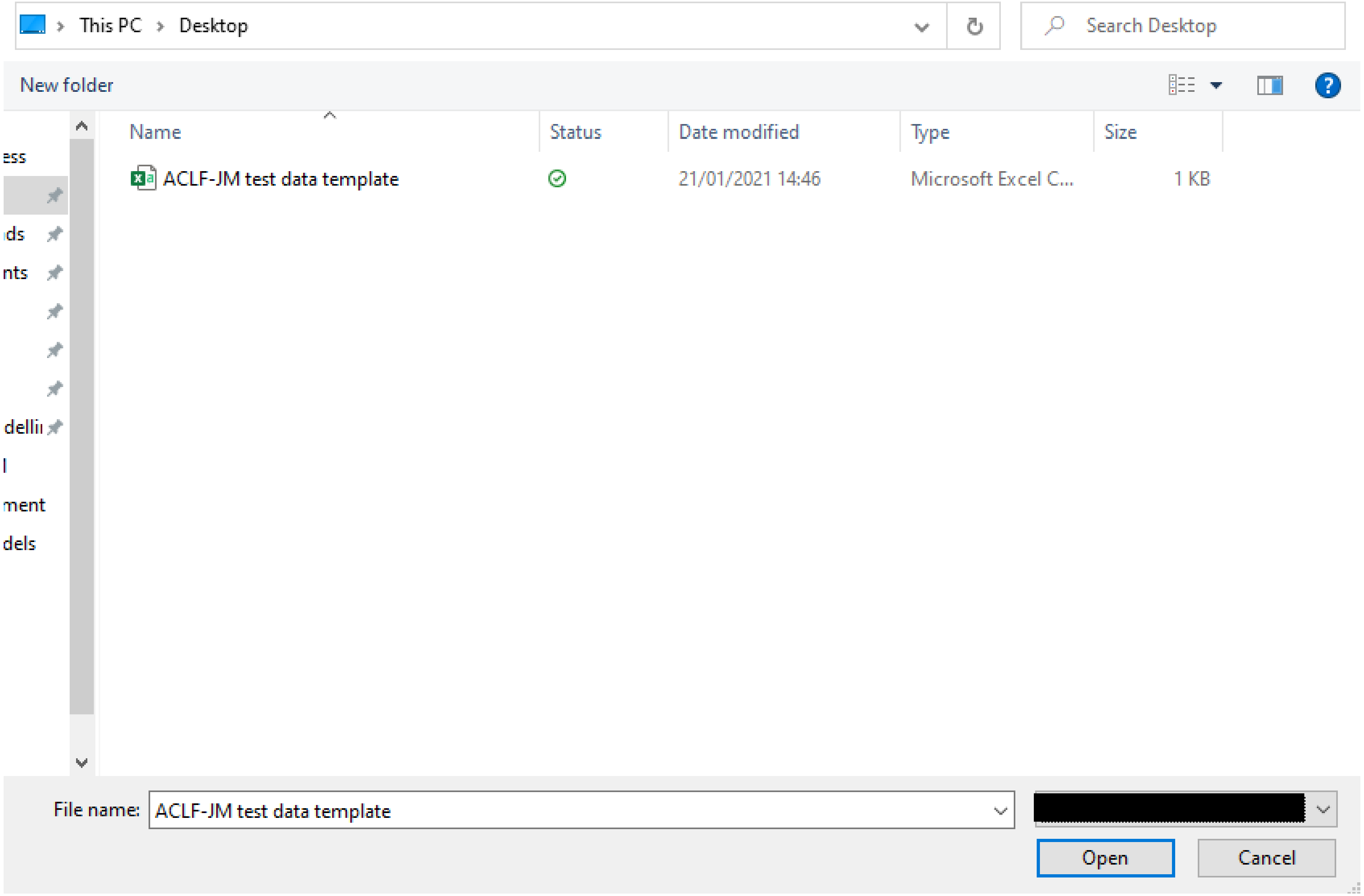
This PC › Desktop
Search Desktop
New folder
Name
Status
Date modified
Type
Size
ACLF-JM test data template
21/01/2021 14:46
Microsoft Excel C...
1 KB
ess
ds
nts
delli
ment
dels
File name:
ACLF-JM test data template
[redacted]
Open
Cancel

The loaded data is previewed in the “Data” tab. Now you can either select a specific prediction interval (e.g. 90 days) or leave it empty. The latter will give survival probabilities over multiple intervals.

Please click the “Survival Probabilities” tab and wait a few seconds for your predictions to load.

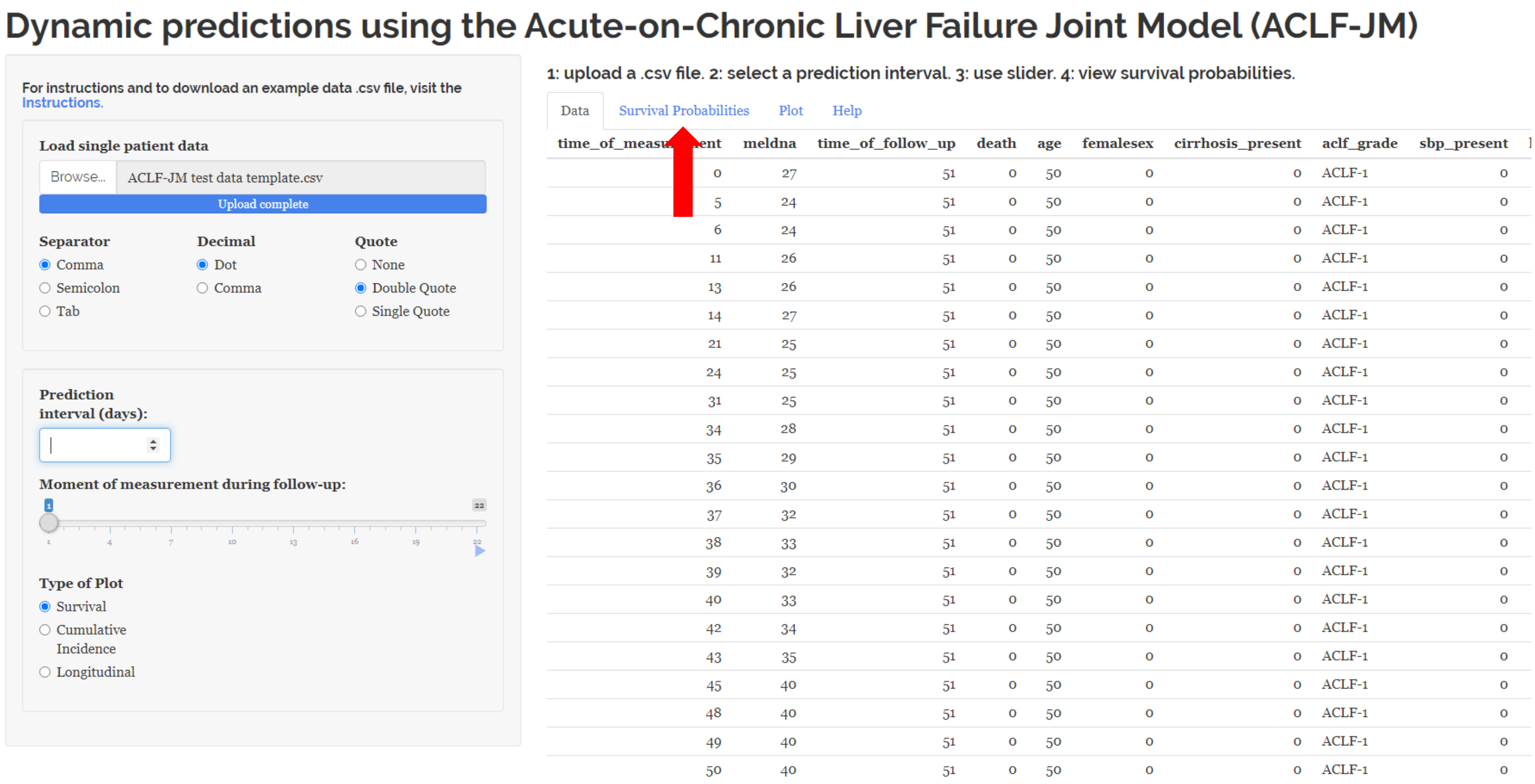

Below, the prediction interval is left empty. Also the slider titled “Moment of measurement during follow-up” is set to the left-most value. The slider summarizes the follow-up period of your patient. The left represents the start of follow-up (day 0) and the right represents the end of follow-up (e.g. day 51). As the slider is set on the left-most value, it shows the expected survival probabilities from day 0.

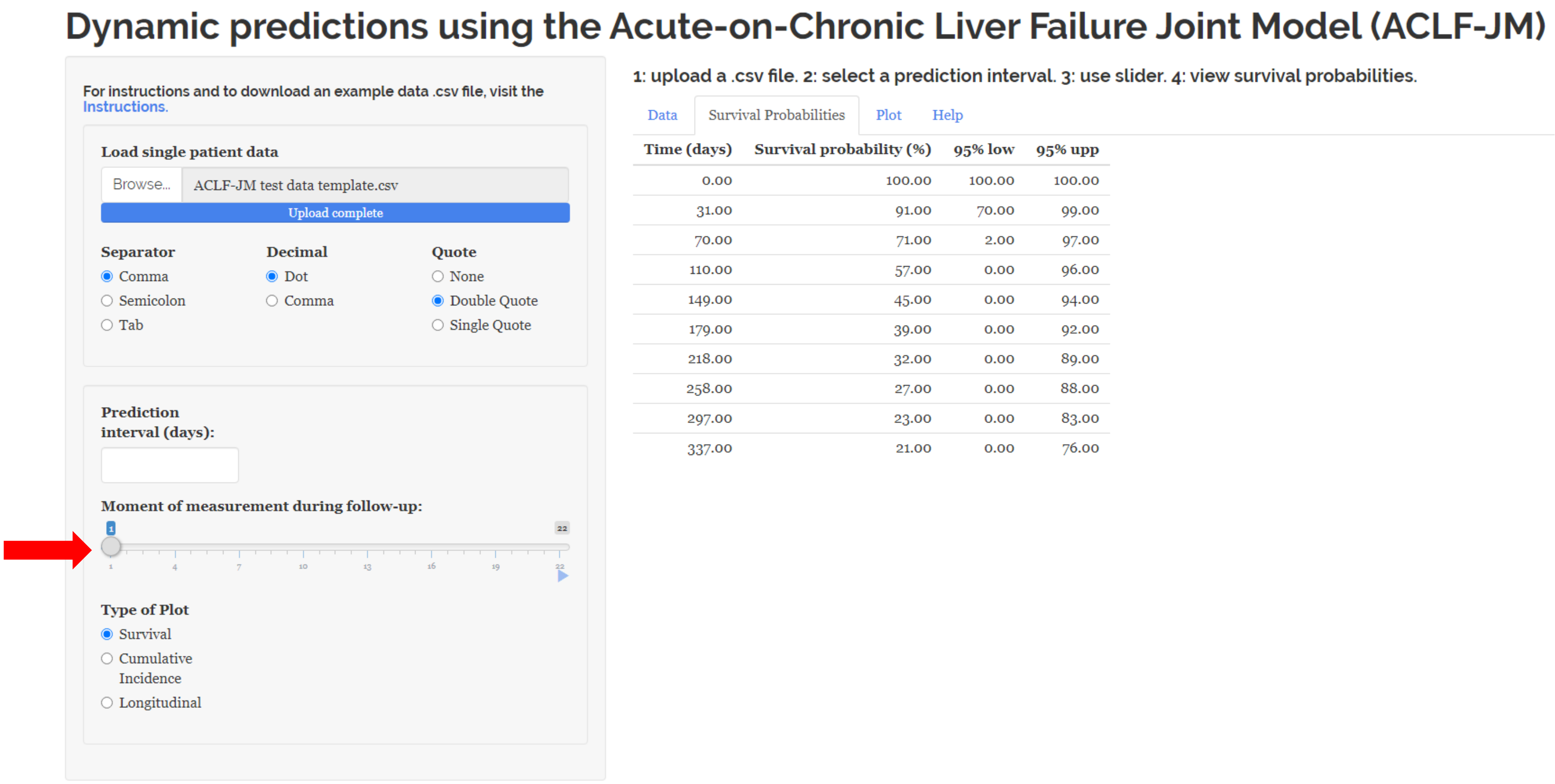

Time (days)	Survival probability (%)	95% low	95% upp
0.00	100.00	100.00	100.00
31.00	91.00	70.00	99.00
70.00	71.00	2.00	97.00
110.00	57.00	0.00	96.00
149.00	45.00	0.00	94.00
179.00	39.00	0.00	92.00
218.00	32.00	0.00	89.00
258.00	27.00	0.00	88.00
297.00	23.00	0.00	83.00
337.00	21.00	0.00	76.00

Sliding the slider to the right will calculate predictions from the last moment of follow-up. In the example below, the patient is still alive at day 50 (100% survival). However, roughly 90 days later, the patient is expected to have been deceased.

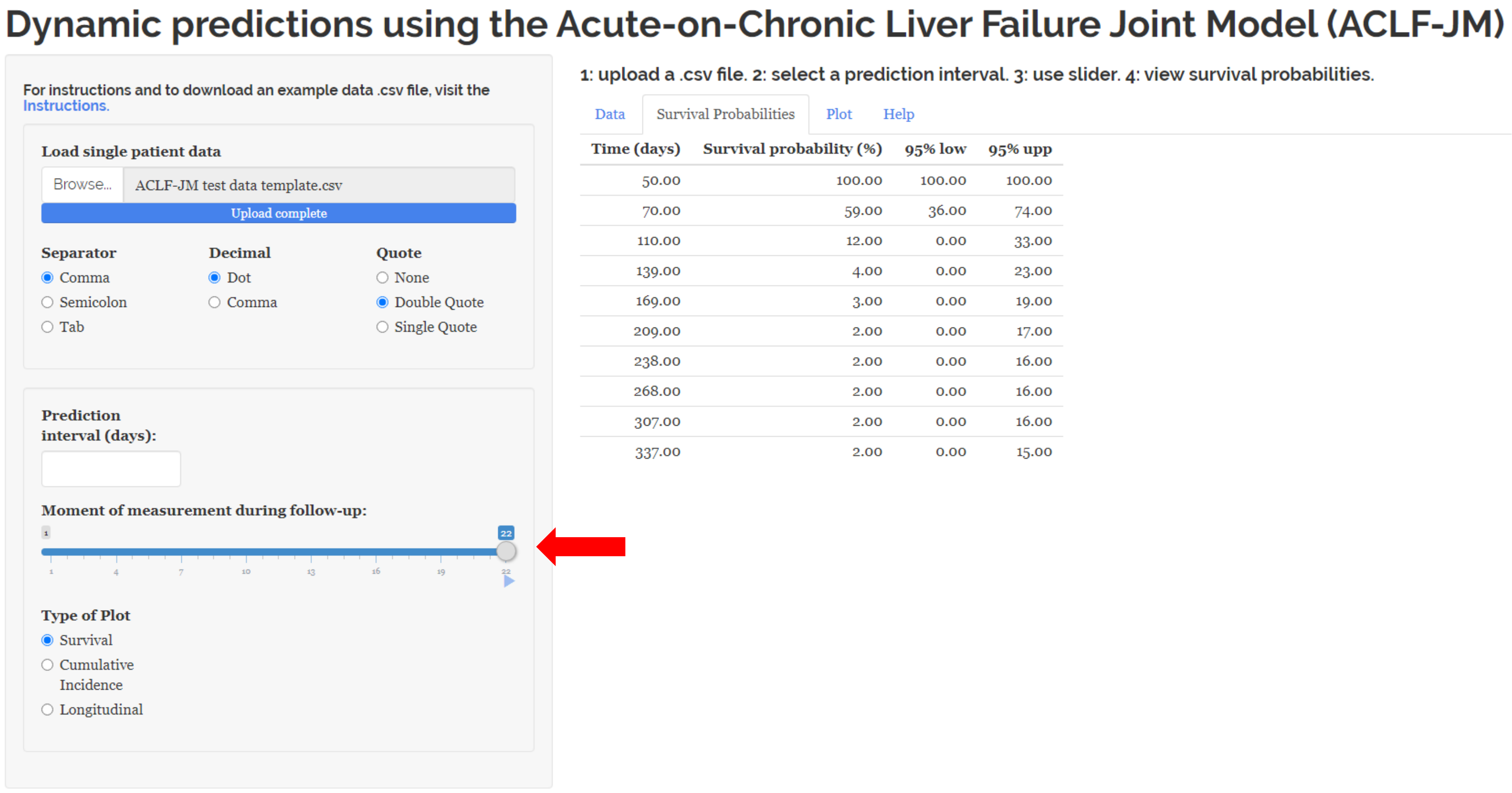

In the “Plot” tab, the survival probabilities and their 95% confidence interval (grey area) are plotted. Please change the slider or press the little “play” button to show the longitudinal disease development (*) and expected survival probabilities.

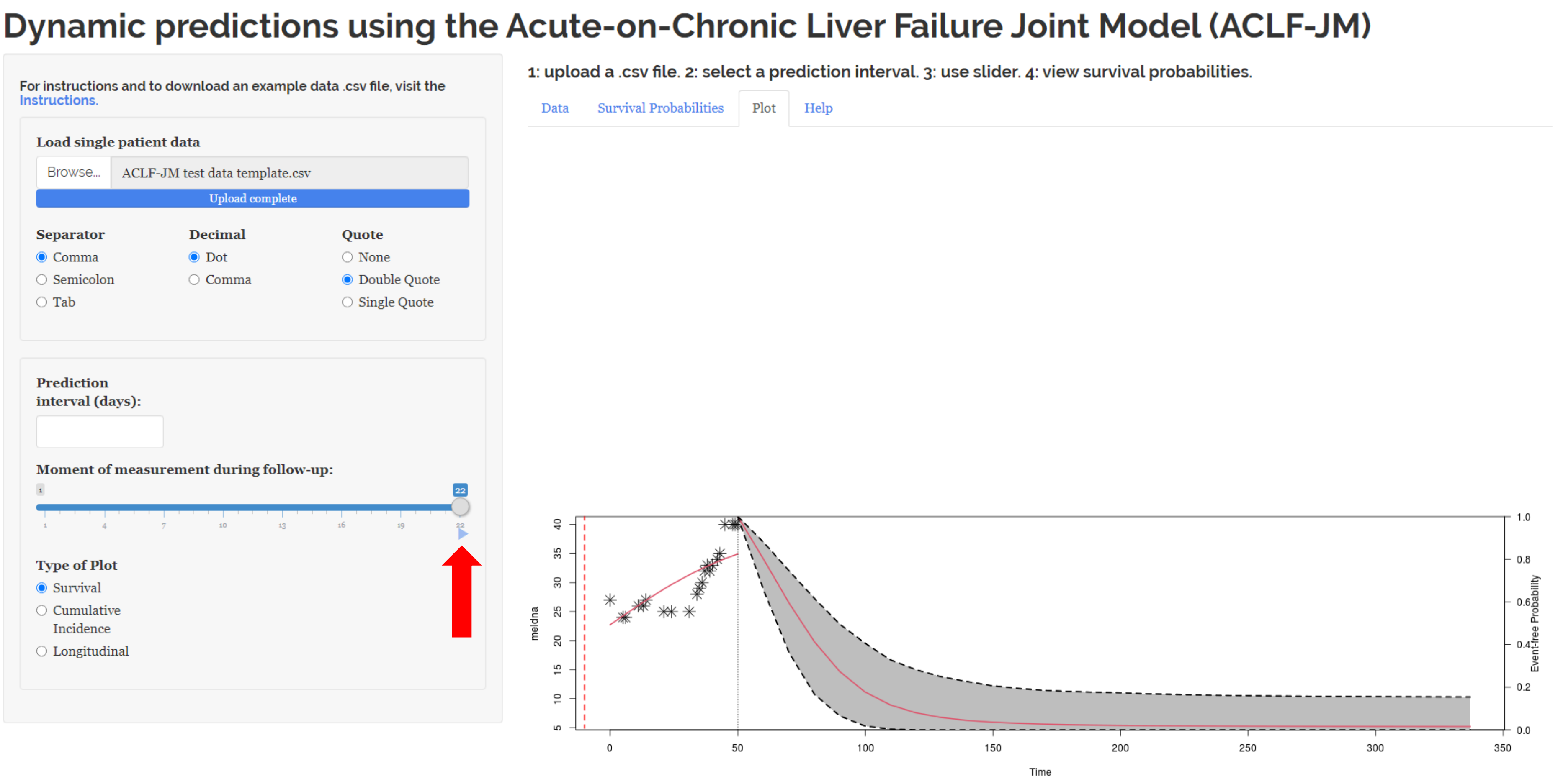

Specifying a prediction interval (e.g. 90 days), will give the calculated survival probabilities for that interval. Once again, you can change the slider to change the moment of follow-up. Below, the slider is set to the right (i.e. the last available measurement) and the predicted 90-day survival probability is 4% for this patient.

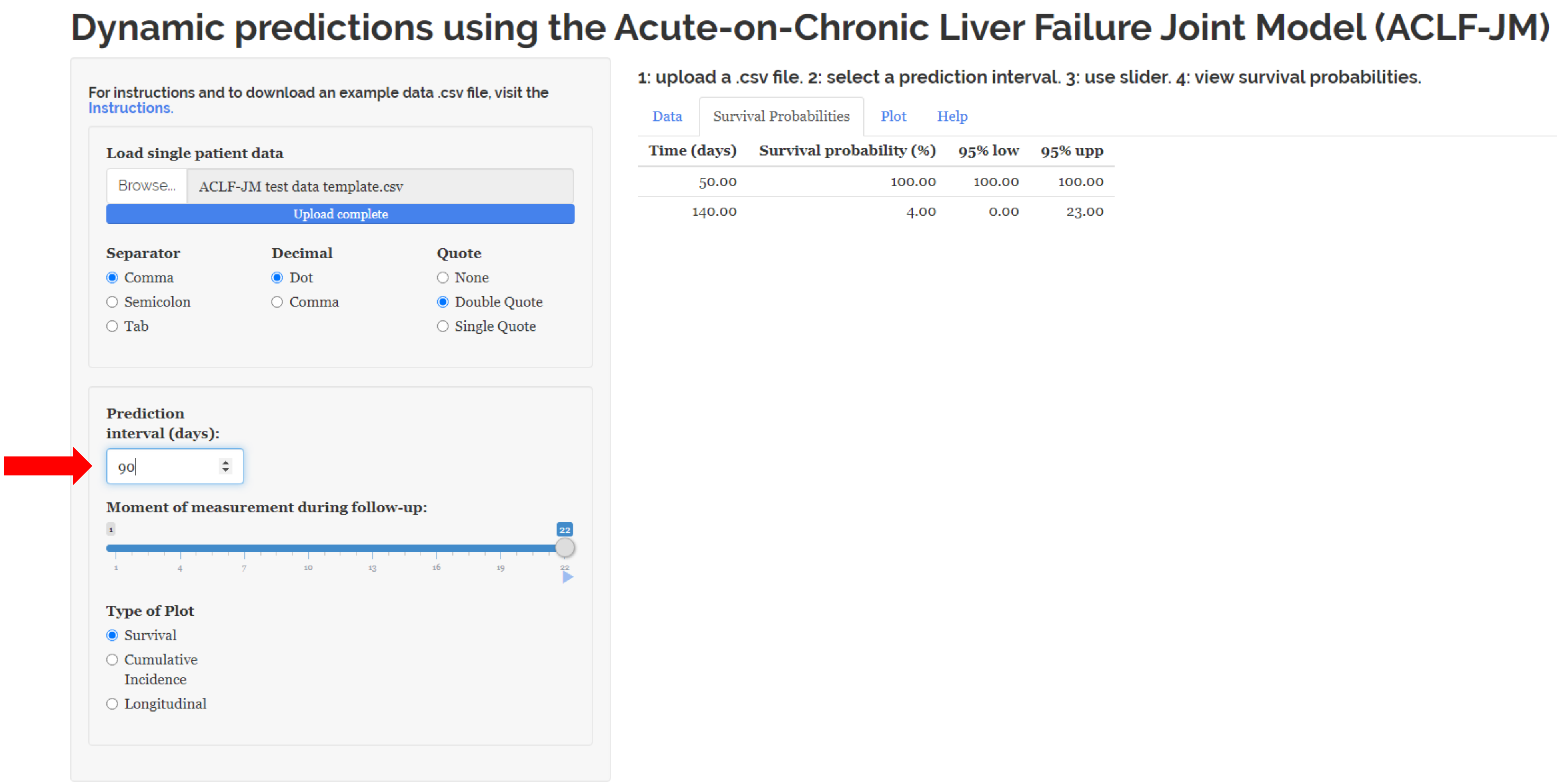

The “Help” tab provides more details and instructions.

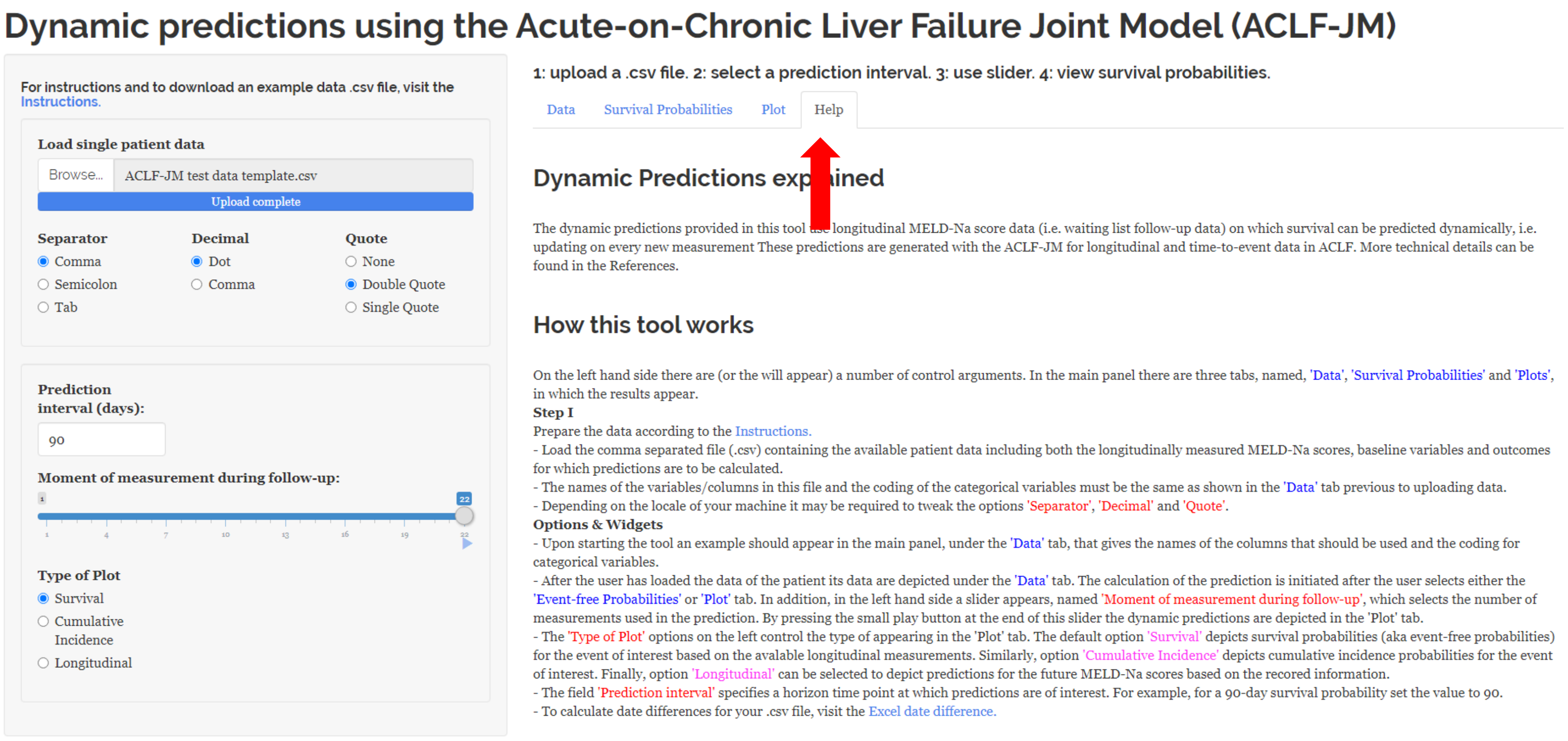

Supplement 3: in-depth study methodology

Statistical analysis – Joint Model construction

Data were randomly split in a training (67%) and a testing (33%) set, for model construction and validation respectively. A non-linear mixed-effect model was constructed, based on repeated measurements of MELD-Na. Mixed-effect models have several advantages: first, they can handle imbalanced data, i.e. a varying number of follow-up measurements per patient (missingness) and time differences between each measurement. Second, they consider in-patient correlation: measurements from one patient are more strongly correlated than measurements from different patients. Third, both the average and individual patient trajectories are modeled simultaneously. Fourth: non-linear developments can be modeled through flexible spline-based estimations of disease. The longitudinal MELD-Na data were corrected for candidate age, sex, life support dependency, presence of bacterial peritonitis, presence of cirrhosis (alcohol-induced, hepatitis-C virus, non-alcoholic steatohepatitis (NASH) or other cirrhosis) and CLIF-C OF score. The used variables were selected a priori, based on clinical relevance and statistical significance in univariate analysis. The mixed-effect JM output was added to the supplement (Table S1), as the natural spline coefficients do not offer an intuitive interpretation. We did however add a plot of the spline-based mixed effect of average MELD-Na development over time (Fig. S5). For the survival prediction part, a Cox proportional hazards model was used. We did not use competing risks analysis, but rather a censorship framework. The reason for this was that a competing risks model should not be used to develop prediction models for allocation priority. To illustrate, consider an environment in which livers are readily available for patients with MELD=40, but only rarely available for patients with MELD=20. A physician might predict, correctly, that a MELD=40

patient has lower risk of dying in the next week because they are almost certain to get a transplant, while the MELD=20 patient is still quite ill and will not receive a liver. However, it would be perverse to use this prediction to determine allocation priority. Changing allocation priority changes the association between MELD and risk of transplant, which changes the association between MELD and risk of mortality in a competing risks framework but not in a censorship framework. Instead, priority should be based on mortality risk _if a transplant is not obtained_, which is properly modeled using censorship. Then, the Cox model (survival component) was jointly-modeled to the mixed-effect model (longitudinal component) with the R JM package.[23] At every moment in time, the JM used both the MELD-Na score and the rate of change in MELD-Na (decrease/increase) for prediction.

Statistical analysis – Joint Model performance

Next, the prediction performance of the JM was compared to the MELD-Na score at various points in time in the separate testing data. To compare results to the CLIF-C OFs performance known from literature, predictions were assessed at baseline and after a follow-up of 48 hours, 7 days and 14 days.[6] Outcomes were 28-day and 90-day survival. A landmark Cox model of MELD-Na scores was fit at the abovementioned times, which used the last available measurement for survival prediction. For the JM, weighted averages of time-dependent AUCs and prediction errors were calculated through Monte Carlo Markov Chain (MCMC) simulations with the JM software.[23] An excellent explanation of time-dependent performance measures for joint models is at http://www.drizopoulos.com/vignettes/dynamic_predictions and shttps://cran.r-project.org/web/packages/JM/JM.pdf. Here from we quote: “Two general approaches have been proposed in the literature to assess predictive performance of survival models, namely, calibration, i.e., how well the model predicts the observed data, and

discrimination, i.e., how well can the model discriminate between patients that had the event from patients that did not.

Discrimination: To take into account the dynamic nature of the longitudinal marker in discriminating between subjects, we focus on a time interval of medical relevance within which the occurrence of events is of interest. In this setting, a useful property of the model would be to successfully discriminate between patients who are going to experience the event within this time frame from patients who will not. To put this formally, as before, we assume that we have collected longitudinal measurements $Yj(t)$ up to time point t for subject j. We are interested in events occurring in the medically-relevant time frame $(t, t + \Delta t]$ within which the physician can take an action to improve the survival chance of the patient. Under the assumed model and the methodology presented in the previous section, we can define a prediction rule using $\pi j(t + \Delta t \mid t)$ that takes into account the available longitudinal measurements $Yj(t)$. In particular, for any value c in $[0,1]$ we can term subject j as a case if $\pi j(t + \Delta t \mid t) \leq c$ (i.e., occurrence of the event) and analogously as a control if $\pi j(t + \Delta t \mid t) > c$. For a randomly chosen pair of subjects $\{j, j'\}$, in which both subjects have provided measurements up to time t, the discriminating capability of the assumed model can be assessed by the area under the receiver operating characteristic curve (AUC), which is obtained for varying c and equals:

$$AUC(t, \Delta t) = \Pr\left[\pi j(t + \Delta t \mid t) < \pi j'(t + \Delta t \mid t) \mid \{T * j \in (t, t + \Delta t]\} \cap \{T * j' > t + \Delta t\}\right]$$

that is, if subject j experiences the event within the relevant time frame whereas subject j' does not, then we would expect the assumed model to assign higher probability of surviving longer than $t + \Delta t$ for the subject who did not experience the event.

The dynamic discrimination index is computed through:

$C_{dyn}^{\Delta t} \ = \int_0^{tmax} AUC(t, \Delta t)\ Pr\{E(t, \Delta t)\}\ dt\ \ /\ \int_0^{tmax} Pr\{E(t, \Delta t)\}\ dt$,

where $AUC(t, \Delta t)$ is defined above, and

$E(t, \Delta t)\ =\ \{T * i\ \in\ (t, t\ +\ \Delta t]\}\ \cap\ \{T * j\ >\ t\ +\ \Delta t\}$,

with i and j denote a randomly selected pair subjects, and $\pi i(t + \Delta t \mid t)$ and $\pi j\ (t + \Delta t \mid t)$ denote the conditional survival probabilities calculated for these two subjects, for different time windows Δt. The upper limit of integral is specified. The integrals in the numerator and denominator are approximated using a 15-point Gauss-Kronrod quadrature rule.

Calibration: The assessment of the accuracy of predictions of survival models is typically based on the expected error of predicting future events. In our setting, and again taking into account the dynamic nature of the longitudinal outcome, it is of interest to predict the occurrence of events at $u > t$ given the information we have recorded up to time t. This gives rise to expected prediction error:

$$PE(u \mid t) = E[L\{Ni(u) - \pi i(u \mid t)\}],$$

where $Ni(t) = I(T * i > t)$ is the event status at time t, $L(\cdot)$ denotes a loss function, such as the absolute or square loss, and the expectation is taken with respect to the distribution of the event times."

Ensuring adequate performance during follow-up is important for possible clinical application. To provide intuitive interpretation of JM AUC and error reduction compared to MELD-Na, the percentage improvement in AUC towards the maximum achievable AUC of 1 was calculated similar to Jalan et al.: $\frac{(AUC_{JM} - AUC_{MELD-Na})}{(1 - AUC_{MELD-Na})} * 100$.[6] However, they presented this AUC improvement as "prediction error improvement", which technically is not correct.

We therefore separately assessed the JM prediction error reduction compared to MELD-Na, with the following calculation: $\frac{(Error_{MELD-Na} - Error_{JM})}{(Error_{MELD-Na})} * \mathbf{100}$.

Statistical analysis – Joint Model dynamic prediction

Finally, individual dynamic predictions were generated. Data from a real ACLF patient (obtained from the testing data) was MCMC simulated and used as input for the trained JM.[23] Plots were created of these dynamic predictions, to show the updating survival estimate for every new available measurement during follow-up. All statistical analyses were performed using R v4.0.0 (R Foundation for Statistical Computing, Vienna, Austria).

Supplement 4: explanation risk calculation figure 1

Consider three patients A, B and C who have been waiting 20 days for LT. A, B and C are identical, except for their MELD-Na scores. Patient A was stable at MELD-Na score 30, i.e. a slope of 0. Patient B started with MELD-Na 20 (baseline) and steadily increased to MELD-Na 25 (day 20), which is a slope of $\frac{5}{20/365} = 91.3$ points per year. Patient C started with MELD-Na 10 at baseline and showed a rapid increase in MELD-Na scores. This results in MELD-Na score 20 at day 20 and a slope of 0.5 MELD-Na per day, which is 182 points per year. The current liver allocation system would give priority to patient A with MELD-Na score 30. However, the JM accounts for past measurements and further discriminates based on slope, i.e. considers that B and C have increasing disease severity and A is stable. Thus, the HR of death for each patient at day 20 can be calculated by multiplying the MELD-Na HR for value and slope (table 2). For patient A: $1.15^{30} * 1.02^{0} = 66.2$, patient B: $1.15^{25} * 1.02^{91.3} = 200.7$ and patient C: $1.15^{20} * 1.02^{182} = 601.4$. So at day 20, the JM would give patient B a $\frac{200.7}{66.2} = 3$ times higher HR compared to patient A and patient C a $\frac{601.4}{66.2} = 9$ times higher risk, indicating that patient C should be prioritized for transplantation.

Fig. Slegends

1. Cumulative incidence plots of waiting list competing risks outcomes. Death rates are lowest in patients without ACLF and most are either removed or transplanted. With increasing ACLF grade, death and transplantation rates increase.
2. Baseline ROC and calibration plots are shown. The ACLF-JM has a significantly (p<0.001) higher 90-day mortality AUC in ACLF patients than MELD-Na, 0.875 (95%CI 0.840-0.909) and 0.780 (0.737-82.3) respectively. The ACLF-JM is also better calibrated than the MELD-Na. This is shown in figure 2-B. The closer the model stays to the diagonal, the better the predicted and observed risks match, i.e. more accurate predictions.
    a. The ROC plot for 90-day mortality prediction in patients with ACLF.
    b. The calibration plot for 90-day mortality prediction in patients with ACLF.
3.
    a. The improvement in AUCs of the ACLF-JM compared to MELD-Na. With increasing ACLF severity, the ACLF-JM AUC improvement increases.
    b. The prediction error improvement of the ACLF-JM compared to MELD-Na. With increasing ACLF disease severity, ACLF-JM prediction error improvement increases.
4. An illustration of the difference between the continuous and flexible ACLF-JM approximated course of development over time (yellow dotted line), versus that of a time-dependent Cox model (red solid lines). The ACLF-JM assumes a continuous, flexible disease development over time. This means that even if values are missing or old, adequate estimates of current disease severity are given. In contrast, for time-

dependent Cox models, the last measurement is linearly carried on forward. With infrequent measurement or missing values, this last observation does not represent the current state of disease. Also, with fast-changing disease severity, Cox models underestimate disease severity because they do not estimate the 'true' underlying developments. The blue arrows point at the moments where the Cox model would underestimate disease severity. Clinically, this could result in underestimation of mortality when evaluation the need for LT in a fast-declining ACLF patient.

5. Spline-based intuitive output, rationale for non-linear model.
6. ROC plot of 90-day survival prediction in patients delisted in the first 28 days of follow-up.

Fig. S1: cumulative incidence plots per ACLF grade

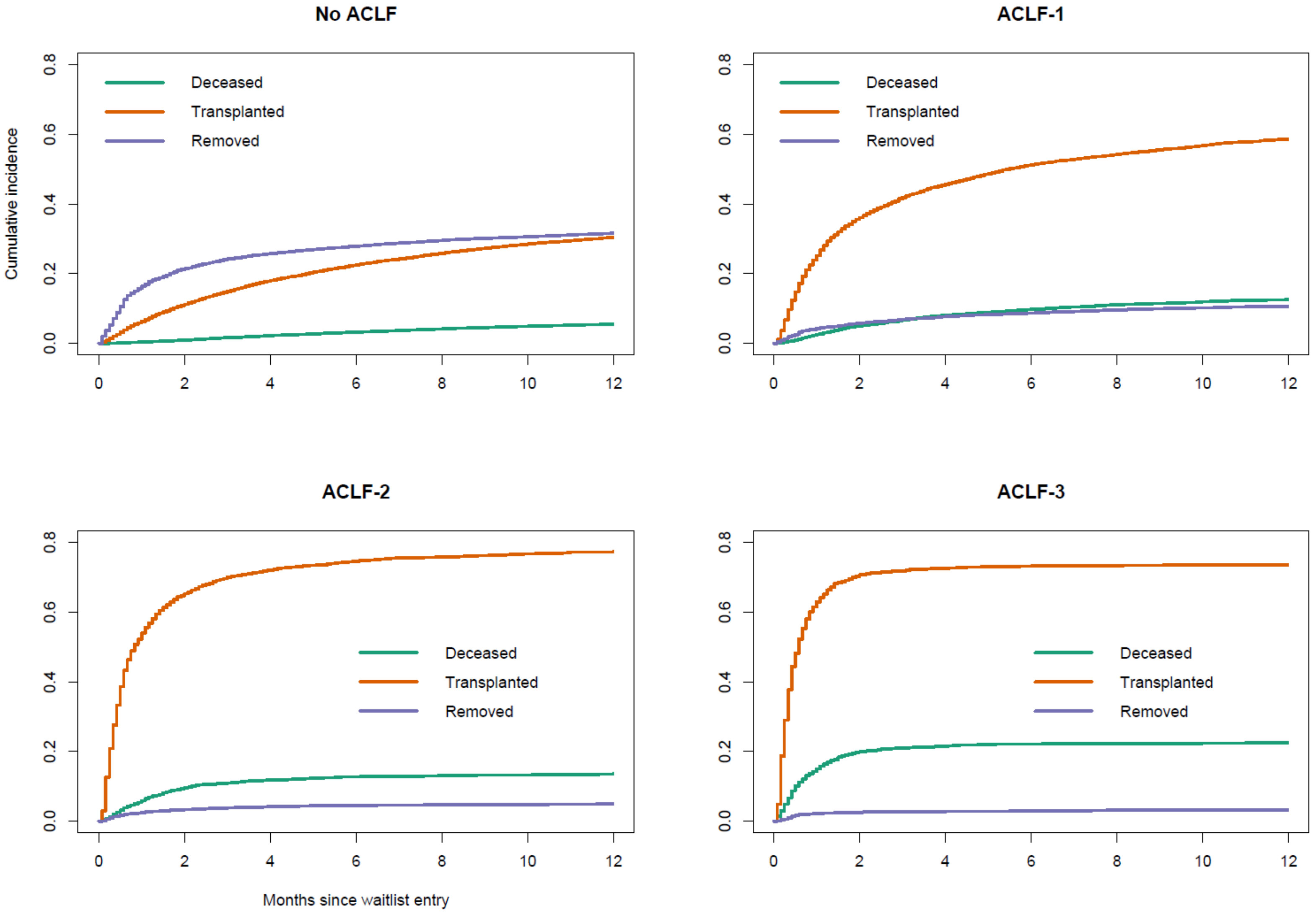

Fig. S2A: ROC plot of the ACLF-JM and MELD-Na

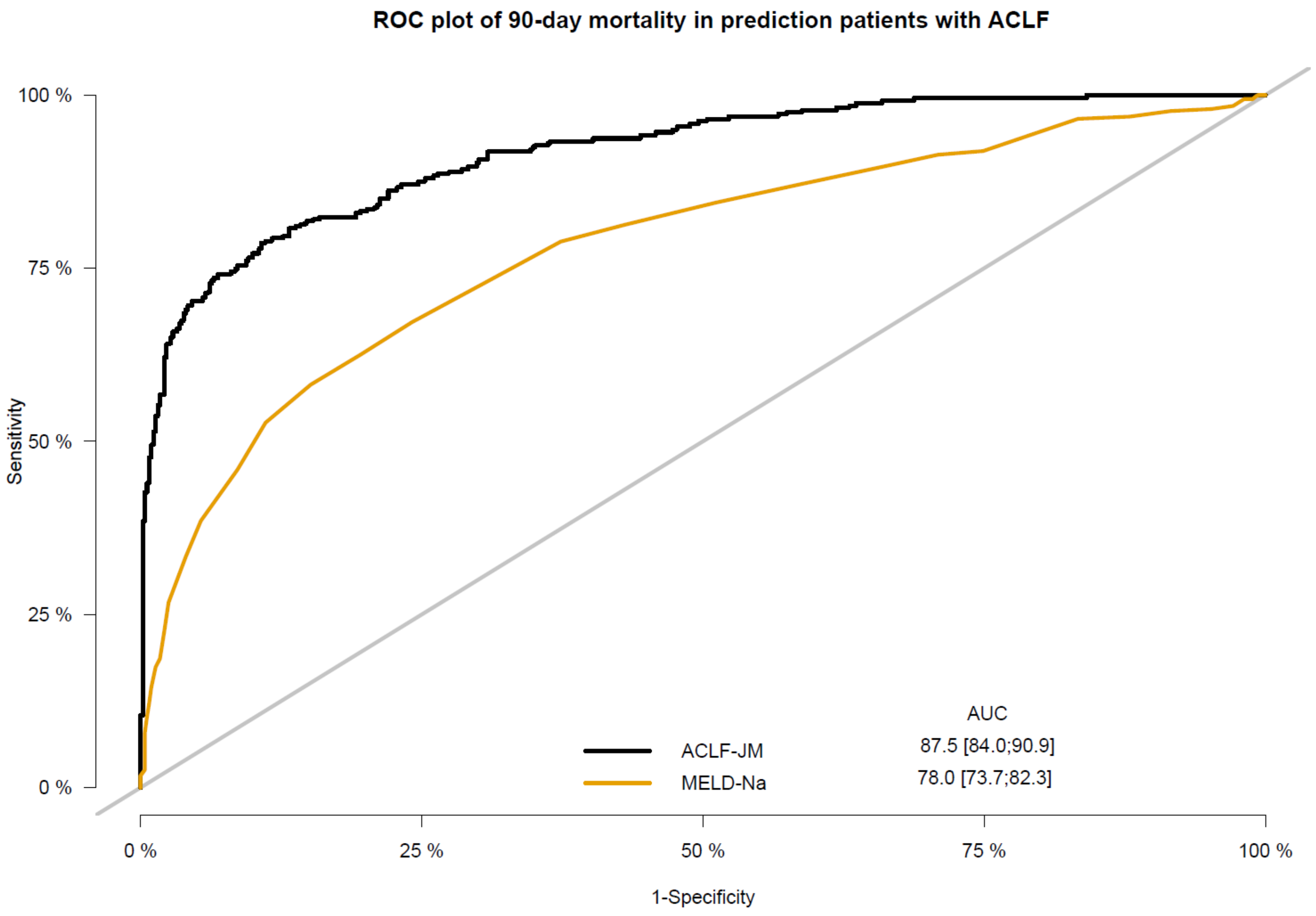

Fig. S2B: Calibration plot of the ACLF-JM and MELD-Na

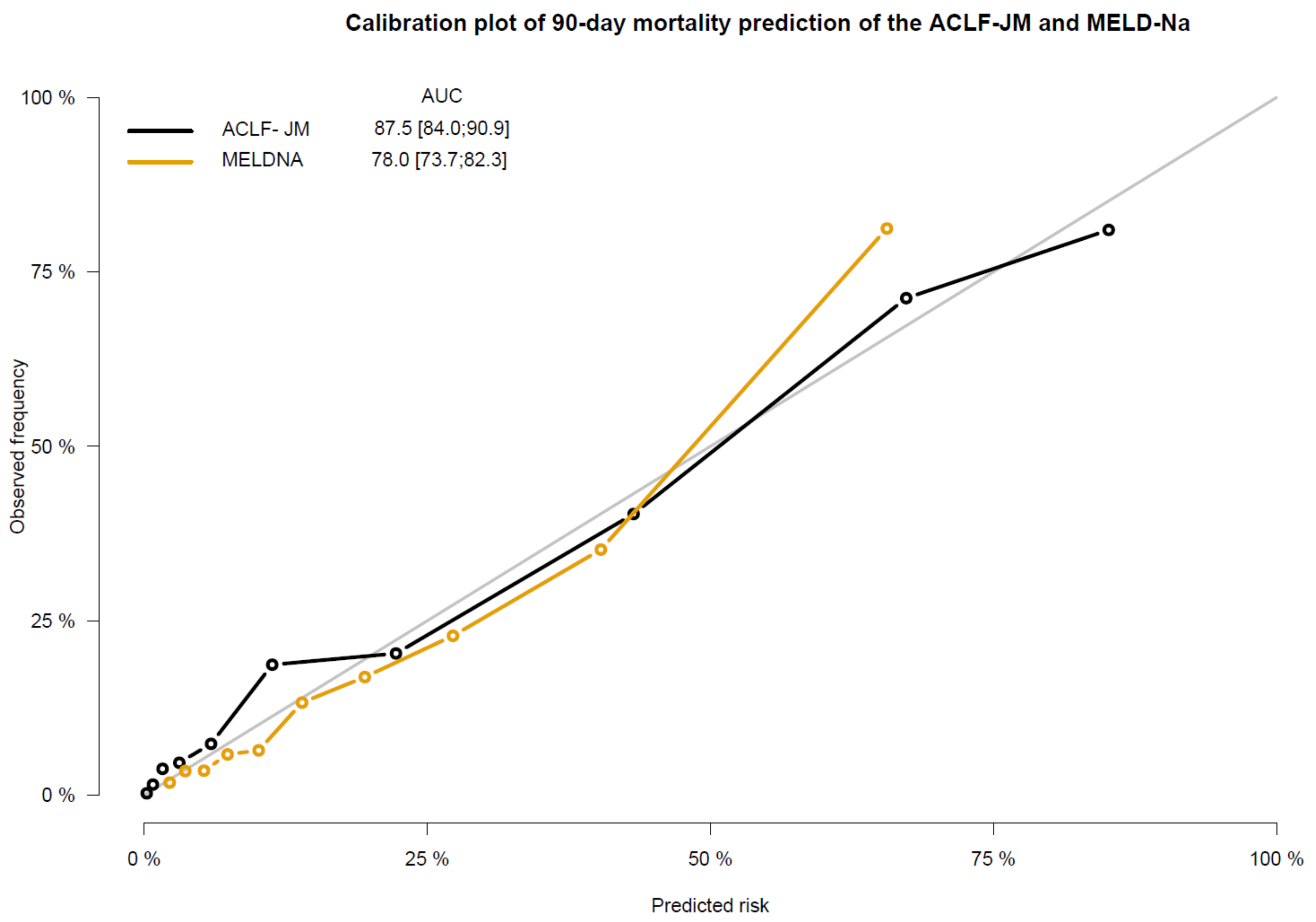

Fig. S3A: AUC improvement in percentages

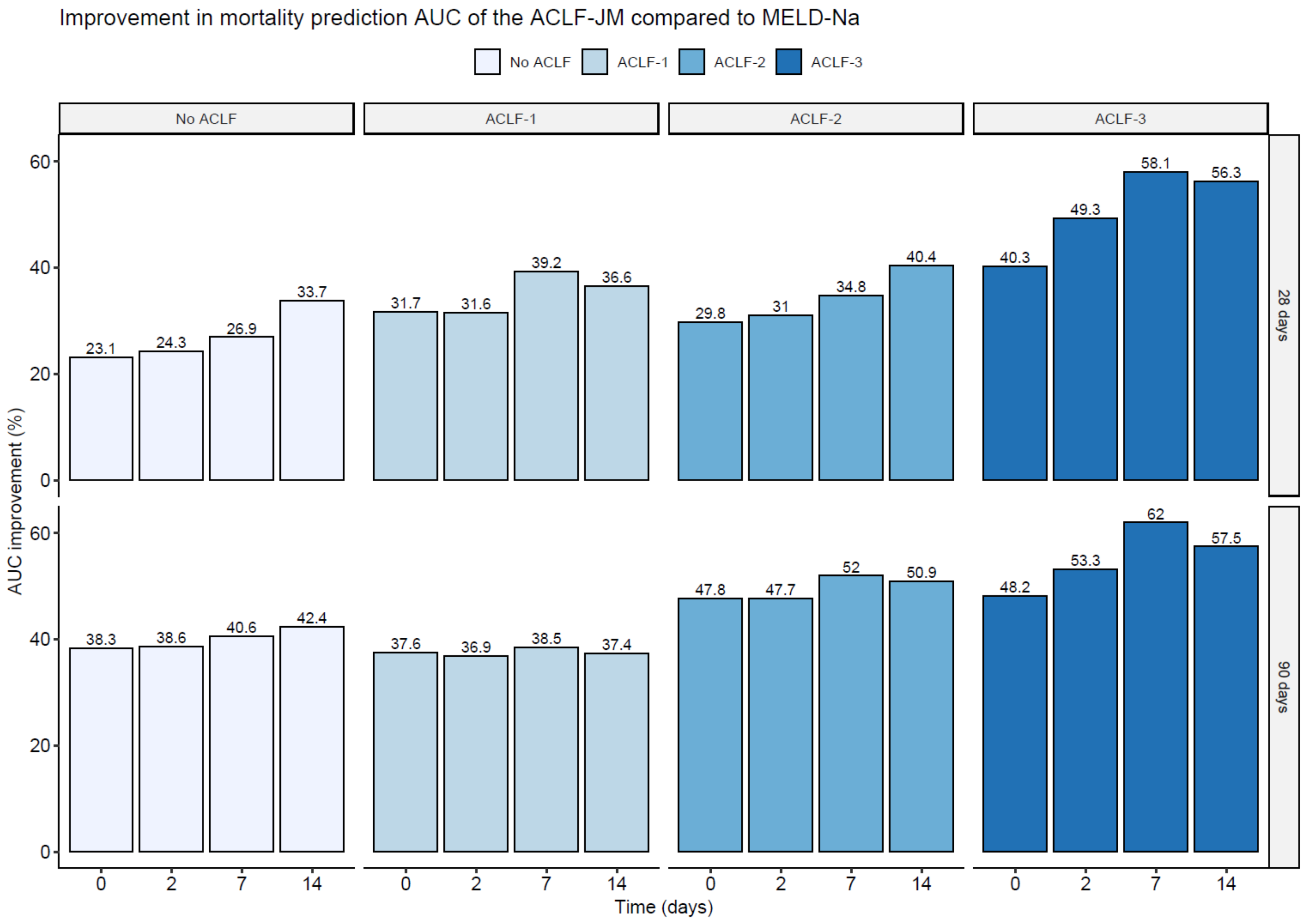

Fig. S3B: prediction errors improvements in percentages

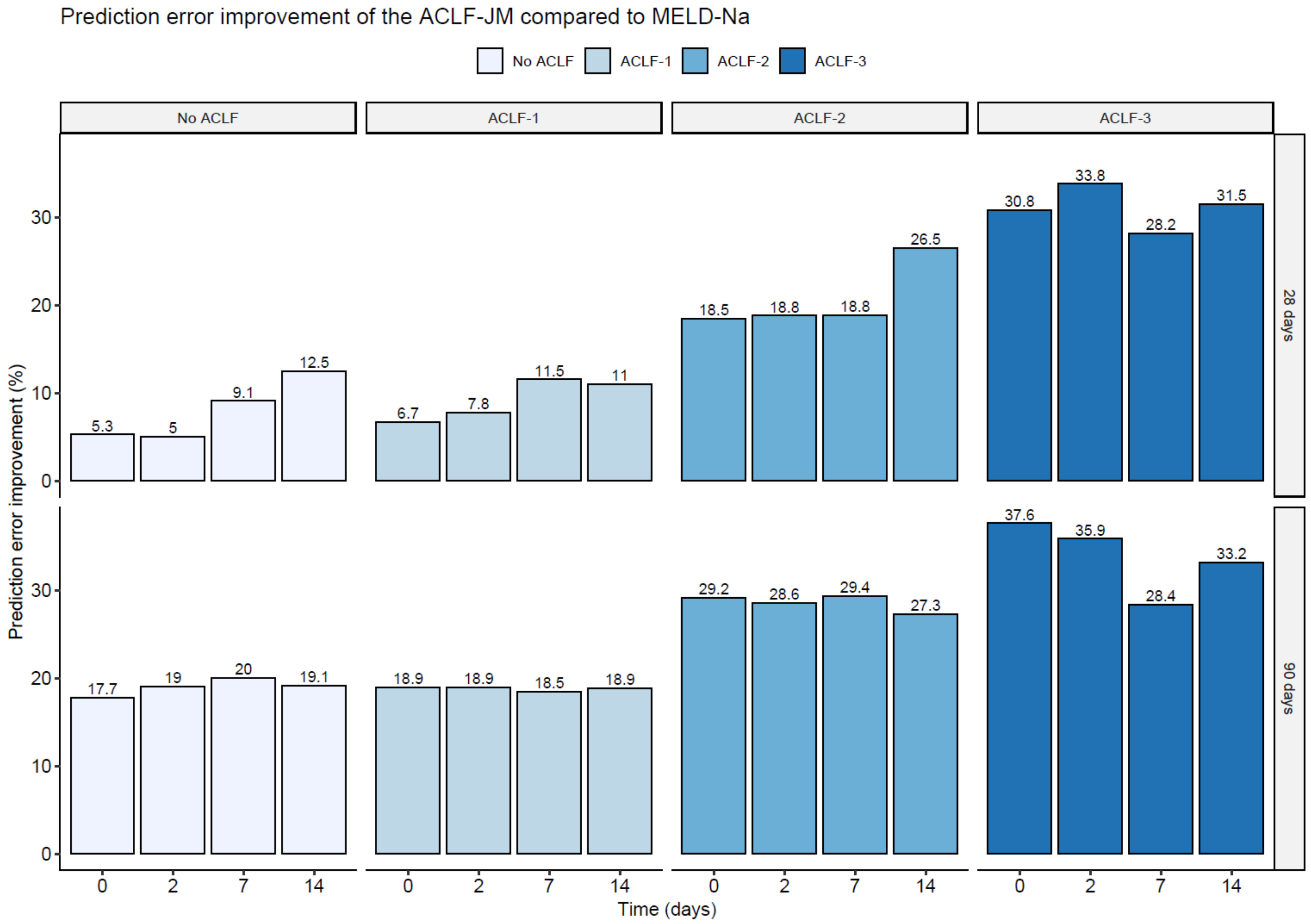

Fig. S4: illustration of model approximation of disease trajectory over time

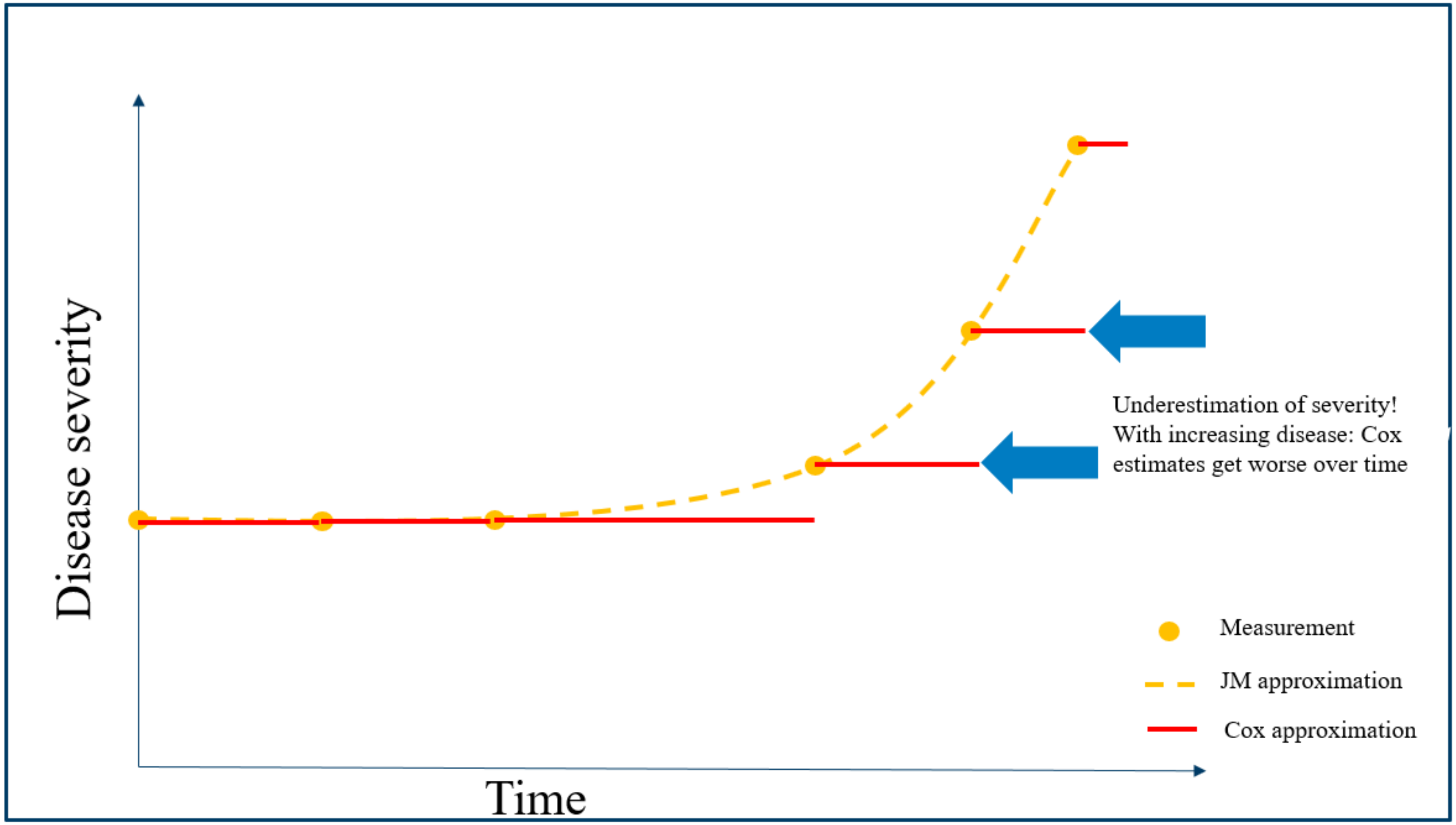

Fig. S5: Spline-based mixed effect plot of the average MELD-Na development over time

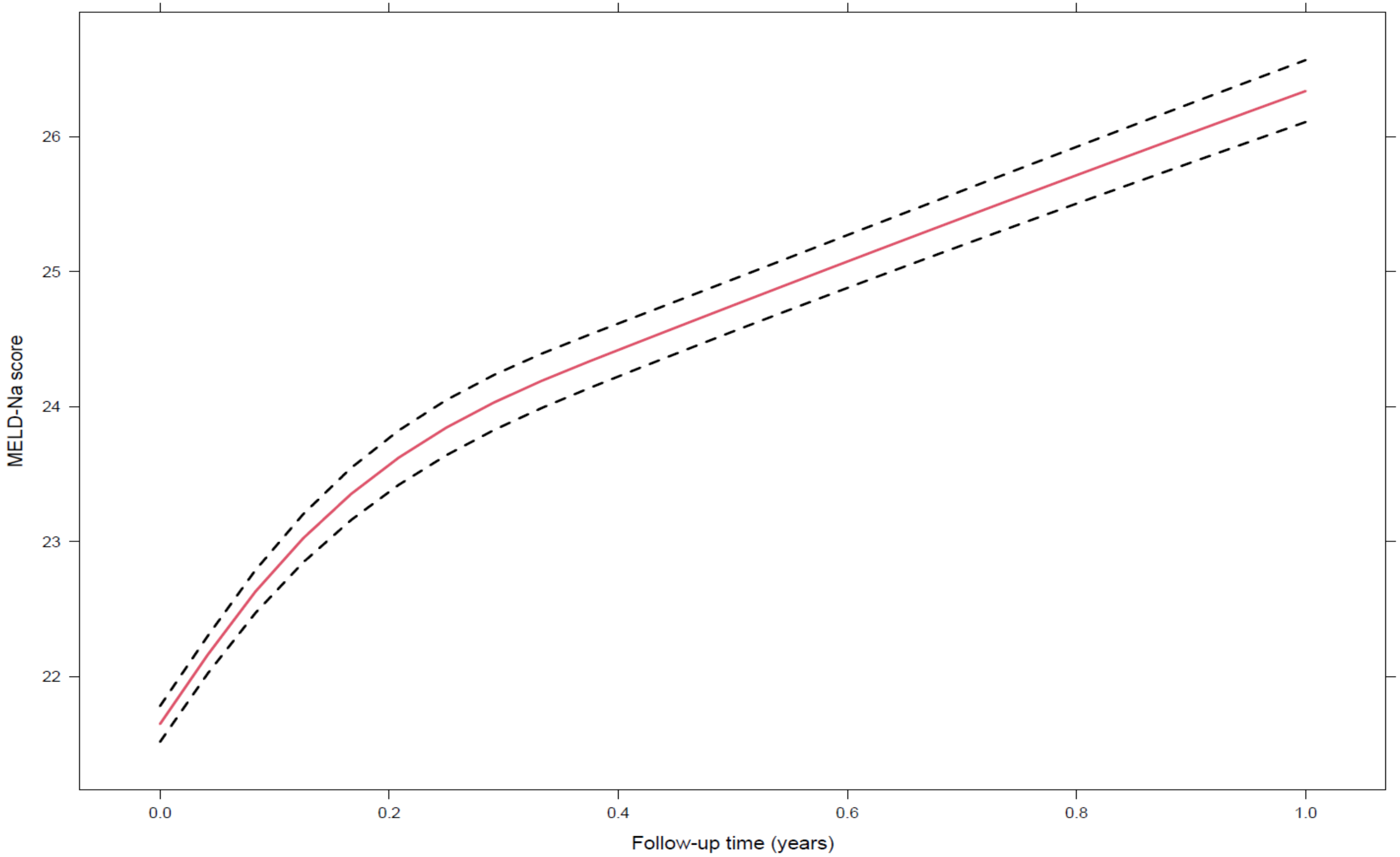

Fig. S6: ROC plot of the ACLF-JM and MELD-Na for delisted patients

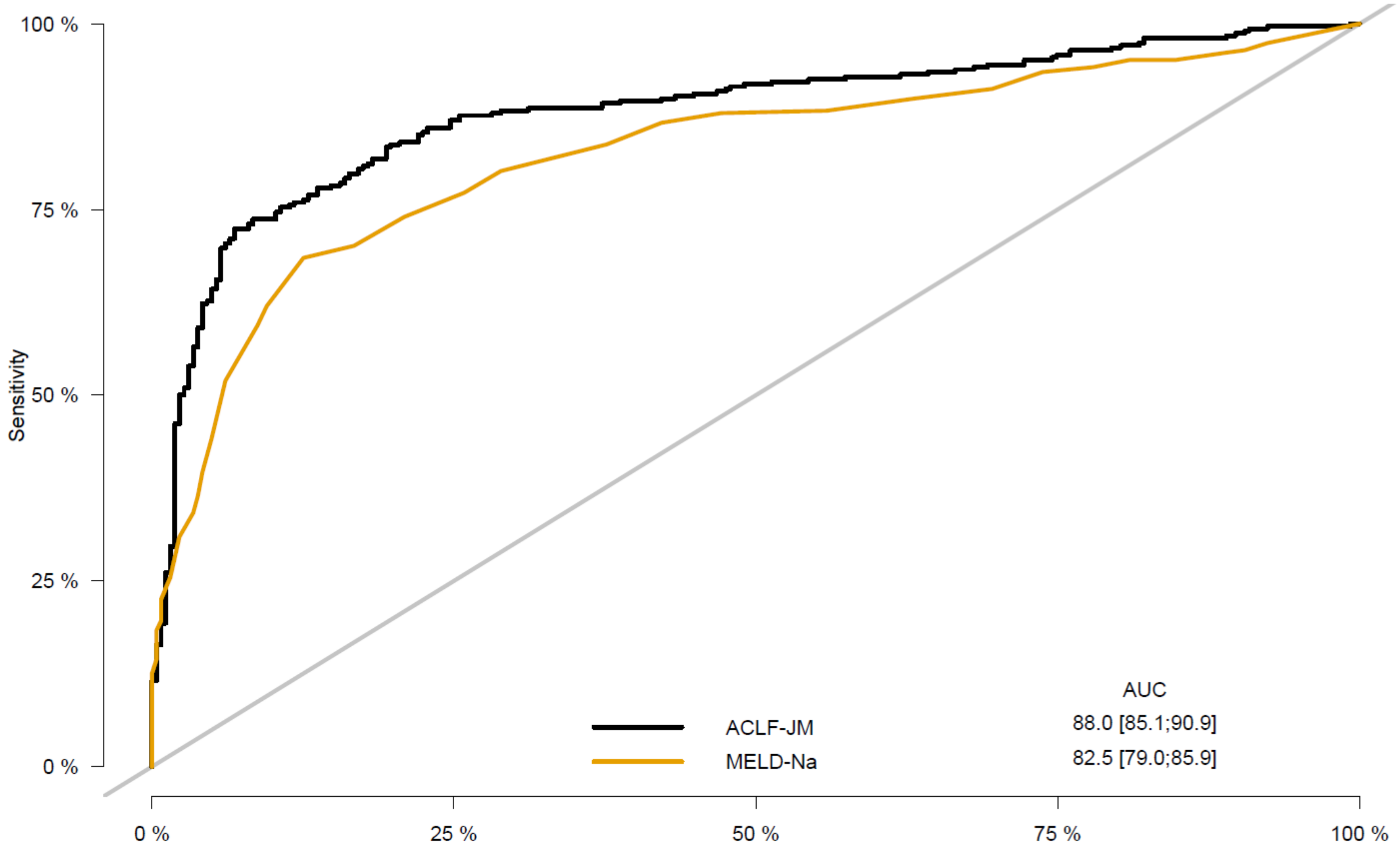

Table S1: mixed effect model output

Spline mixed-effect model output					
	Value	Std.Error	DF	t-value	p-value
(Intercept)	16.05	0.27	145979	60.46	***
ns(years, df=3)1	5.25	0.26	145979	20.24	***
ns(years, df=3)2	10.56	0.25	145979	42.95	***
ns(years, df=3)3	11.44	0.49	145979	23.47	***
age10	-0.55	0.04	20448	-12.57	***
femalegender	0.40	0.10	20448	4.20	***
aclfACLF-1	10.83	0.13	20448	85.33	***
aclfACLF-2	17.18	0.15	20448	111.05	***
aclfACLF-3	21.16	0.21	20448	98.93	***
Cirrhosis (HCV, NASH, ALD, other)	2.85	0.12	20448	24.50	***
life_sup	-2.93	0.26	20448	-11.22	***
sbp	2.14	0.15	20448	13.96	***

Table S2: List of candidate variables investigated for the joint model construction

CAN_ABO	Patient/s Blood Type
CAN_AGE_AT_LISTING	Calculated Candidate Age at Listing
CAN_AGE_IN_MONTHS_AT_LISTING	Calculated Candidate Age in Months at Listing
CAN_ARTIFICIAL_LI	Life Support://Artifical Liver
CAN_ASCITES	Ascites
CAN_BACTERIA_PERIT	Spontaneous Bacterial Peritonitis
CAN_BMI	BMI:
CAN_CTP_SCORE	Candidate/s CTP score (used for MAOB Liver Variance
CAN_DGN	Primary Diagnosis
CAN_DGN2	Secondary Diagnosis
CAN_DGN_OSTXT	Primary Diagnosis/Specify
CAN_DIAB	Diabetes
CAN_DIAB_TY	Diabetes
CAN_DIAL	Dialysis
CAN_ECMO	Life Support: ECMO
CAN_ENCEPH	Encephalopathy
CAN_ETHNICITY_SRTR	SRTR Patient Ethnicity
CAN_FUNCTN_STAT	Patient/s Functional Status
CAN_GENDER	Patient/s Gender
CAN_HGT_CM	Candidate/s Height (stored in cm)
CAN_IABP	Life Support: IABP
CAN_INIT_SRTR_LAB_MELD	First SRTR MELD/PELD given
CAN_IV_INOTROP	Life Support: IV Inotropes
CAN_LAST_ALBUMIN	Candidate Last Albumin (used for MELD)
CAN_LAST_ASCITES	Candidate Last Ascites (used for MELD)
CAN_LAST_BILI	Candidate Last Bilirubin (used for MELD)
CAN_LAST_DIAL_PRIOR_WEEK	Last non-blank val. of dialysis within prior week
CAN_LAST_ENCEPH	Candidate Last Encephalopathy (used for MELD)
CAN_LAST_INR	Candidate Last INR (used for MELD)
CAN_LAST_SERUM_CREAT	Candidate Last Serum Creatinine mg/dl (used for MELD)
CAN_LAST_SERUM_SODIUM	Candidate Last Serum Sodium (used for MELD)

CAN_LAST_SRTR_LAB_MELD	Last SRTR MELD/PELD given
CAN_LIFE_SUPPORT	Patient on Life Support
CAN_LIFE_SUPPORT_OTHER	Life Support: Other Mechanism
CAN_MALIG	Any previous Malignancy
CAN_MALIG_TY	Previous Malignancy Type(s)
CAN_MOST_RECENT_CREAT	Most Recent Absolute Creatinine
CAN_MOST_RECENT_HGT_CM	Candidate/s most recent Waitlist Height in centimeter
CAN_MOST_RECENT_WGT_KG	Candidate/s most recent Waitlist Weight in kilograms
CAN_MUSCLE_WASTING	Marked Muscle Wasting
CAN_NEOPLASM	Neoplasm
CAN_PREV_ABDOM_SURG	Previous Upper Abdominal Surgery
CAN_PREV_TX	Previous Transplants
CAN_RACE	Patient/s Race
CAN_TIPSS	History of TIPSS
CAN_TOT_ALBUMIN	Total Serum Albumin
CAN_TOT_BILI	Total Bilirubin (IN Pediatric Only)
CAN_VARICEAL_BLEEDING	Variceal Bleeding within Last Two Weeks
CAN_VENTILATOR	Life Support: Ventilator
CAN_WGT_KG	Candidate/s Weight in kilograms

Table S3: AUCs stratified per ACLF grade

Mortality prediction AUC of the ACLF-JM versus the MELD-Na in patients with ACLF, at baseline and during follow-up												
	ACLF-1				ACLF-2				ACLF-3			
**28-day mortality**	JM	95% CI	MELD-Na	95% CI	JM	95% CI	MELD-Na	95% CI	JM	95% CI	MELD-Na	95% CI
Baseline	0.817	0.771-0.862	0.732	0.681-0.783	0.809	0.749-0.870	0.728	0.664-0.792	0.764	0.665-0.863	0.605	0.496-0.714
48 hours	0.816	0.770-0.862	0.731	0.680-0.782	0.813	0.750-0.875	0.729	0.663-0.796	0.779	0.672-0.887	0.564	0.442-0.685
7 days	0.825	0.778-0.872	0.712	0.656-0.768	0.807	0.739-0.874	0.704	0.629-0.778	0.792	0.663-0.921	0.504	0.363-0.645
14 days	0.818	0.763-0.872	0.713	0.648-0.779	0.820	0.738-0.903	0.698	0.608-0.788	0.780	0.626-0.935	0.497	0.334-0.659
	ACLF-1				ACLF-2				ACLF-3			
**90-day mortality**	JM	95% CI	MELD-Na	95% CI	JM	95% CI	MELD-Na	95% CI	JM	95% CI	MELD-Na	95% CI
Baseline	0.829	0.791-0.868	0.726	0.679-0.773	0.859	0.802-0.916	0.730	0.651-0.808	0.841	0.634-0.949	0.693	0.453-0.933
48 hours	0.824	0.786-0.863	0.721	0.674-0.769	0.862	0.804-0.920	0.736	0.656-0.815	0.843	0.636-0.950	0.664	0.424-0.905
7 days	0.824	0.784-0.864	0.714	0.666-0.763	0.856	0.796-0.917	0.700	0.614-0.786	0.853	0.643-0.963	0.613	0.408-0.818
14 days	0.809	0.764-0.854	0.695	0.642-0.748	0.836	0.766-0.907	0.666	0.567-0.766	0.844	0.613-0.976	0.633	0.423-0.844

ACLF: acute-on-chronic liver failure, AUC: area under receiver operator curve

JM: joint model, MELD-Na: model for end-stage liver disease sodium score

Table S4: Advantages and disadvantages of the MELD-Na and ACLF-JM

Cox MELD-Na

Pros	Cons
Easy to interpret	Uses one moment/measurement in time to assess disease severity
Calculated quickly	Ignores previous measurements
	Assumes linear disease development
	Predicts survival based on population averages

Joint model

Pros	Cons
Adequately handles complex follow-up data (missing, irregularly measured)	Takes longer to compute
Considers different correlation of measurements within or between patients	Statistically complex
Assesses both development over time and time-to-event data	
Uses all available measurements	
Updates predictions for every new measurement, i.e. accumulating evidence	
Can model both linear and non-linear disease development	
Predicts both on population and individual patient level	
Can simulate individual patient data to calculate personalized predictions	

[end of manuscript: this page is left empty]
