# Supplementary material for: Development and validation of a dynamic survival prediction model for patients with acute-on-chronic liver failure
Source: JHEP Rep. 2021 Sep 29;3(6):100369. doi: 10.1016/j.jhepr.2021.100369 (PMC8570961; doi:10.1016/j.jhepr.2021.100369)
Supplement: Multimedia component 2 [file mmc2.pdf]

JOURNAL
OF HEPATOLOGY

# Journal of Hepatology

## CTAT methods

Tables for a "Complete, Transparent, Accurate and Timely account" (CTAT) are now mandatory for all revised submissions. The aim is to enhance the reproducibility of methods.

- Only include the parts relevant to your study
- Refer to the CTAT in the main text as 'Supplementary CTAT Table'
- Do not add subheadings
- Add as many rows as needed to include all information
- Only include one item per row

**If the CTAT form is not relevant to your study, please outline the reasons why:**

This study is observational in nature and the data was obtained retrospectively.

### 1.1 Antibodies

| Name | Citation | Supplier | Cat no. | Clone no. |
|---|---|---|---|---|
| N/A | N/A | N/A | N/A | N/A |

### 1.2 Cell lines

| Name | Citation | Supplier | Cat no. | Passage no. | Authentication test method |
|---|---|---|---|---|---|
| N/A | N/A | N/A | N/A | N/A | N/A |

### 1.3 Organisms

| Name | Citation | Supplier | Strain | Sex | Age | Overall n number |
|---|---|---|---|---|---|---|
| N/A | N/A | N/A | N/A | N/A | N/A | N/A |

### 1.4 Sequence based reagents

| Name | Sequence | Supplier |
|---|---|---|
| N/A | N/A | N/A |

### 1.5 Biological samples

| Description | Source | Identifier |
|---|---|---|
| N/A | N/A | N/A |

### 1.6 Deposited data

| Name of repository | Identifier | Link |
|---|---|---|
| N/A | N/A | N/A |

### 1.7 Software

| Software name | Manufacturer | Version |
|---|---|---|
| N/A | N/A | N/A |

### 1.8 Other (e.g. drugs, proteins, vectors etc.)

| | | |
|---|---|---|
| N/A | N/A | N/A |
| N/A | N/A | N/A |

### 1.9 Please provide the details of the corresponding methods author for the manuscript:

B.F.J. Goudsmit, MD
Division of Transplantation, Department of Surgery, Leiden University Medical Centre
Address: Albinusdreef 2, 2333 ZA Leiden, The Netherlands.
e-mail: b.f.j.goudsmit@lumc.nl

### 2.0 Please confirm for randomised controlled trials all versions of the clinical protocol are included in the submission. These will be published online as supplementary information.

N/A
